# EIF4A3-Induced Exosomal circLRRC8A Alleviates Granulosa Cells Senescence Via the miR-125a-3p/NFE2L1 axis

**DOI:** 10.1007/s12015-023-10564-8

**Published:** 2023-05-27

**Authors:** Jie Xing, Mengxue Zhang, Shijie Zhao, Mingjun Lu, Li Lin, Lu Chen, Wujiang Gao, Wenxin Li, Junyu Shang, Jiamin Zhou, Xiaolan Zhu

**Affiliations:** 1 Reproductive Medicine Center, The Fourth Affiliated Hospital of Jiangsu University, Zhenjiang, China; 2 Department of Central Laboratory, The Fourth Affiliated Hospital of Jiangsu University, Zhenjiang, China; 3 Reproductive Sciences Institute, Jiangsu University, Zhenjiang, China

**Keywords:** Premature ovarian failure, CircLRRC8A, MiR-125a-3p, NFE2L1, EIF4A3, Exosomes

## Abstract

Premature ovarian failure (POF) is an important cause of female infertility and seriously impacts the physical and psychological health of patients. Mesenchymal stromal cells-derived exosomes (MSCs-Exos) have an essential role in the treatment of reproductive disorders, particularly POF. However, the biological function and therapeutic mechanism of MSCs exosomal circRNAs in POF remain to be determined. Here, with bioinformatics analysis and functional assays, circLRRC8A was found to be downregulated in senescent granulosa cells (GCs) and acted as a crucial factor in MSCs-Exos for oxidative damage protection and anti-senescence of GCs in vitro and in vivo. Mechanistic investigations revealed that circLRRC8A served as an endogenous miR-125a-3p sponge to downregulate NFE2L1 expression. Moreover, eukaryotic initiation factor 4A3 (EIF4A3), acting as a pre-mRNA splicing factor, promoted circLRRC8A cyclization and expression by directly binding to the LRRC8A mRNA transcript. Notably, EIF4A3 silencing reduced circLRRC8A expression and attenuated the therapeutic effect of MSCs-Exos on oxidatively damaged GCs. This study demonstrates a new therapeutic pathway for cellular senescence protection against oxidative damage by delivering circLRRC8A-enriched exosomes through the circLRRC8A/miR-125a-3p/NFE2L1 axis and paves the way for the establishment of a cell-free therapeutic approach for POF. CircLRRC8A may be a promising circulating biomarker for diagnosis and prognosis and an exceptional candidate for further therapeutic exploration.

**Graphical Abstract:**

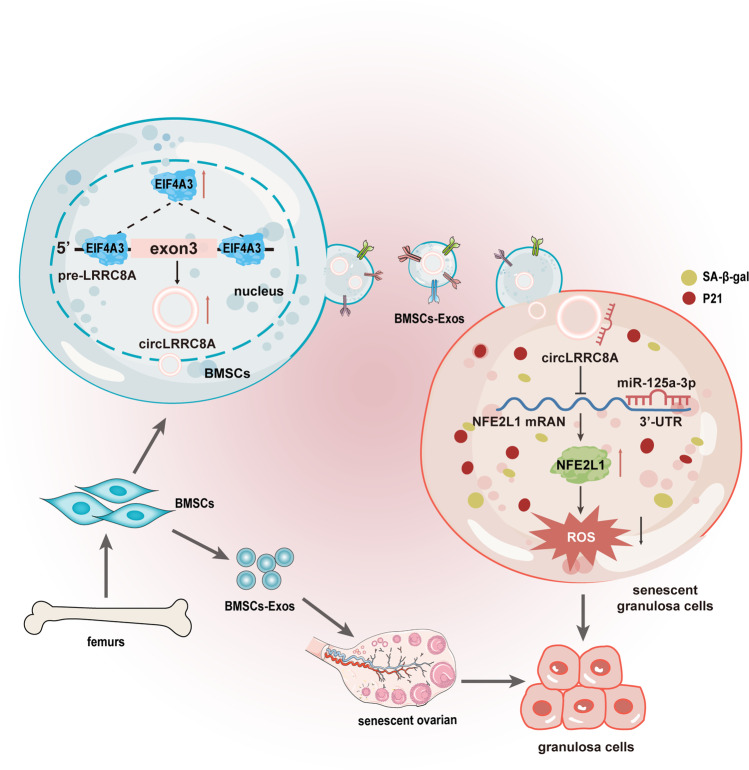

**Supplementary Information:**

The online version contains supplementary material available at 10.1007/s12015-023-10564-8.

## Introduction

Premature ovarian failure (POF) is a mysterious and complicated disease characterized by hypoestrogenism, hypergonadotropinism, and amenorrhea that contributes to female infertility and premenopausal syndrome [1]. Although the etiology of POF remains obscure, a poor primordial follicle pool or accelerated follicle depletion are considered the major mechanisms of POF [2]. It has been suggested that granulosa cells (GCs) not only provide nutrients to oocytes but also protect oocytes from oxidative stress during follicle development [3]. Accumulated reactive oxygen species (ROS) levels in GCs are not a simple consequence of cellular senescence but also a trigger and accelerator of ovarian aging. ROS-mediated oxidative stress results in GCs apoptosis to promote abnormal follicular development [4]. These observations point to the idea that GCs dysfunction may contribute to the pathogenesis and development of POF [5].

Although long-term hormone replacement therapy is the main therapeutic method for POF, it is difficult to restore typical ovarian function [6]; moreover, it may increase the risk of endometrial, breast, and ovarian cancer [7]. As a result, new treatments are needed to restore ovarian function in patients with POF. Recently, mesenchymal stromal cells (MSCs) therapy has been reported to be a promising approach to treating POF [8]. However, the risk of tumorigenicity and immune rejection of transplantation is not avoided in MSCs-based therapies [9, 10]. Exosomes are nanoscale biological vesicles that are released into the surrounding body fluid after the fusion of multivesicular bodies with the plasma membrane [11], which with a diameter between 30 and 150 nm that mediate intercellular communication by delivering various biomolecules, including proteins, mRNAs, and noncoding RNAs [12, 13]. An increasing number of studies have demonstrated that MSCs-derived exosomes can replicate the anti-inflammatory, anti-apoptotic, pro-angiogenic, and anti-fibrotic effects of their parent cells and are considered a substitute for cell-based therapies [14]. In addition, their lower tumorigenic risk, superior immune tolerance, and superior stability compared with their parent cells make them an attractive option in regenerative medicine [10, 15, 16].

Circular RNA (circRNA) is a novel endogenous noncoding RNA whose structure comprises covalently closed loops without 5′ end caps and 3′ terminal poly(A) tails. Due to these unique structural conformations, circRNAs can resist exonuclease and RNase R degradation and are more stable than their cognate linear transcripts [17, 18]. The biogenesis of circRNA is regulated by specific cis-acting elements and trans-acting factors [19]. Several circRNAs are abundant and evolutionarily conserved and exert important biological functions by acting as microRNA or protein inhibitors (‘sponges’) [20, 21], by regulating protein function or by being translated themselves [22]. According to this conservative property, circRNA has become a potential therapeutic agent and biomarker in early clinical diagnosis and biological research of aging-related diseases [23]. CircPVT1 sponges let-7 and prevents proliferating fibroblast senescence [24]. Recently, studies have illustrated that circRNAs are abundant and stable in exosomes compared to the producer cells and can transfer biological activity after the exosomes are taken up by neighboring cells [25]. For example, exosomal circRNA_0000253 promotes intervertebral disc degeneration by adsorbing miRNA-141-5p and downregulating SIRT1 [26]. The functions and applications of exosomal circRNAs in POF diagnosis and treatment need to be further explored.

In this study, we investigated the effects of BMSCs-derived exosomes containing circLRRC8A on the regulation of oxidative damage in GCs. To further understand the regulatory mechanism, we identified the miRNA binding partners for the circRNAs and their targets. In addition, molecules promoting circLRRC8A biosynthesis in GCs were identified. Furthermore, we clarified the possibility and feasibility of circLRRC8A as a diagnostic marker and promising therapeutic target for POF patients.

## Materials and Methods

### Cells Culture and Transfection

All cell lines, including HEK293 and KGN cells, were purchased from Shanghai Jihe Biotechnology Co., LTD (Shanghai, China). The HEK293 cells were cultured in RPMI 1640 medium (Gibco, Carlsbad, CA, USA). The KGN cells were cultured in DMEM/F12 medium (Gibco). All the cell lines were supplemented with 10% fetal bovine serum (Gibco, USA) and 1% penicillin-streptomycin at 37 °C and with 5% CO2 in a humidified atmosphere. CircLRRC8A siRNA (si-circLRRC8A), EIF4A3-specific siRNA, miR-125a-3p mimics, anti-miR-125a-3p, si-NFE2L1 and their negative controls siRNA (si-NC) were purchased from Gene Pharma (Shanghai, China). CircLRRC8A-specific over-expression plasmid and negative control plasmid were purchased from GeneChem (Shanghai, China). HEK293 cells and KGN cells were seeded in a six-well plate with a density of 1 × 10^6^ cells, incubated overnight, were then transfected with the related reagents using Lipofectamine 2000 (Thermo Fisher Scientific, USA) as the manufacturer’s instruction.

### Data Acquisition and Processing

The POF-related circRNA microarray dataset (GSE97193) and miRNA microarray dataset (GSE63737) were downloaded from Gene Expression Omnibus (GEO, http://www.ncbi.nlm.nih.gov/geo/). GSE97193 contained granulosa cell samples obtained from IVF patients of 3 women with advanced age (AA, ≥ 38 years) and 3 matched women with young age (YA, ≤ 30 years). GSE63737 contained follicular fluids samples (*n* = 10) from follicles containing MII oocytes which were allocated into two groups according to the patient’s age: advanced age (AA; >36 years old) (*n* = 5) and young age (YA; <36 years old) (*n* = 5).

The probe IDs of the original data were converted to official gene symbols by using data tables of microarray platforms with R software. Raw expression values were log 2 transformed with the Aaffy package encoded by R. Finally, gene expression values were normalized using the normalize Between Arrays function of the R package limma.

### RNA Extraction, Treatment with RNase R, Reverse Transcription, and Quantitative Real-Time PCR (qRT-PCR)

Total RNA was extracted from all cell lines using the TRIzol reagent (Invitrogen, MA, USA). The nuclear and cytoplasmic fractions were extracted using PARIS™ Kit (Thermo Fisher, MA, USA). Two milligrams of total RNA were incubated with or without RNase R (BioVision, M1228-500). The RNA was reverse transcripted into cDNA using FastQuant RT Kit (Tiangen, China). Target RNAs were amplified on a 7500 Real-Time PCR system (Applied Biosystem, USA) using 2X TaqMan Fast qPCR Master Mix (Sangon Biotech, China). GAPDH was selected as an internal control. The relative quantitative values were determined by using the 2 − ΔΔCT method. Related primers are listed in Supplementary Table 1.

### Western Blot

Approximately 2 × 10^6^ treated cells were lysed using RIPA buffer (Solarbio, china) to extract proteins. Protein lysates were separated by 10% SDS-PAGE gels and then transferred onto a PVDF membrane (Merck KGaA, Germany). The membranes were blocked in 5% milk at room temperature for 2 hours and were then incubated with primary antibodies at 4 °C overnight, including anti-CD9 (Abcam, ab236630, 1:1000), anti-CD63 (Abcam, ab134045, 1:1000), anti-EIF4A3 (Abcam, ab180573,1:1000), anti-NFE2L1 (Proteintech,17,062-1-AP,1:1000), anti-P21 (CST, #2947, 1:1000), and anti-GAPDH (Proteintech, #10494–1-AP, 1:5000) antibodies. Finally, protein bands were incubated in peroxidase-coupled avidin goat anti-rabbit IgG (Cell Signaling Technology, MA, USA) at room temperature for 1 hour.

### ROS Levels Detection

Cellular oxidative stress in KGN cells were examined by a ROS assay Kit (Beyotime, China), which sets DCFH-DA as the probe. KGN cells (2 × 10^6^) after treatment were washed with PBS and stained by DCFH-DA (10 μM) at 37 °C for 20 minutes. The fluorescence images of DFC were taken using fluorescence microscope.

### Senescence-Associated β-Galactosidase Activity

Senescence β-Galactosidase Staining Kit (Beyotime, #C0602) was used to measure senescence in KGN cells. KGN cells (2 × 10^6^) were washed with PBS and then were fixed with β-galactosidase fixing solution for 15 min at room temperature. KGN cells were washed three times and then incubated with freshly prepared senescence-associated β-galactosidase activity (SA-β-gal) staining solution at 37 °C overnight.

### RNA-Binding Protein Immunoprecipitation (RIP)

RIP experiments were performed with the help of a Magna RIP RNA-Binding Protein immunoprecipitation Kit (Millipore, Billerica, MA, USA). KGN cells (1 × 10^7^) were lysed by 200 μl RIP lysis buffer with 1 μM RNase inhibitors. The cell lysates were incubated with 50 μl protein A/G beads coated with an antibody against 5 μg EIF4A3 or IgG (negative control).

Finally, qRT-PCR was used to measure EIF4A3-associated RNA enrichment.

### RNA Pull-Down Assays

The biotinylated miR-125a-3p probe and biotinylated negative control (NC) probe were synthesized by Genepharm (Shanghai, China). HEK293 cells (1.5 × 10^7^) were transfected by biotinylated miR-125a-3p probe or its NC probe and lysed by 500 μl lysis Buffer (Thermo Scientific, USA). The cell lysate was incubated with 50 μl streptavidin magnetic beads (Invitrogen, Grand Island, NY, USA) for one night at 4 °C. Finally, RNA was isolated from RNA complex bounded to the beads and valued by qRT-PCR assays.

### Dual Luciferase Reporter Assays

To examine the binding ability between circLRRC8A and miR-125a-3p. Approximately 1 × 10^5^ HEK-293 T cells were seeded in a 24-well plate for 24 h before transfection. The cells were co-transfected with a mixture of luciferase reporter vectors (pmirGLO) containing circLRRC8A-miR-125a-3p binding sequences or mutant sequences and miRNA mimics (20 nM). After 24 h, the luciferase activity was measured using a dual luciferase reporter assay system (Promega, Madison, WI, USA) according to the manufacturer’s protocol. To determine the direct binding between miR-125a-3p and NFE2L1. The 3’UTR sequence of NFE2L1 was cloned into the pcDNA3.0 vector. Next, the miR-125a-3p mimic or NC was co-transfected with a wild-type vector or mutant vector. The relative luciferase value was also detected by a dual luciferase reporter assay system (Promega, Madison, WI, USA).

### Isolation and Culture of BMSCs

BMSCs were obtained from the bone marrow of 5 weeks old female SD rats, isolated as described previously [16], and cultured in DMEM medium with 10% FBS. The culture medium was changed every 3 days. P3-P6 cells were collected for further study.

### Exosome Extraction, Identification, and Labeling

When BMSCs reached 80% confluence, P3-P6 BMSCs were cultured in DMEM medium with 10% exosome-free FBS. After 48 h, the supernatant was collected, centrifuged at 2000×g for 20 min to remove cellular debris, and then concentrated through the ultrafiltration device (UFC900396, Millipore, USA) at 2000×g for 30 min at 4 °C. Finally, the concentrated solution of supernatant was mixed with total exosome isolation kit (Umibio, UR52121) in a ratio of 1:4. The mixture of concentrated solution and reagent were incubated at 4 °C for 2 hours and then centrifuged at 10,000×g for 1 hour. Finally, the exosome pellet was resuspended in PBS stored at −80 °C.

Transmission electron microscopy (TEM) and nanoparticle tracking analysis (NTA) were used to detect the morphology and size of exosomes. Exosomal surface markers including CD9 and CD63 were identified by western blot assays.

Purified exosomes (150 μg) were incubated with 5 μM Dil (Beyotime, # C1036) for 20 min at 37 °C. Unbound dye was removed by centrifugation at 12,000×g for 30 min using the ultrafiltration device (UFC900396, Millipore, USA), and then the pellet was washed twice with precooled PBS. The obtained Dil-Exos were resuspended in PBS and stored at −80 °C.

### Induction of GCs Senescence In Vitro, BMSCs and Exosomes Treatment

KGN cells (1 × 10^6^) were seeded into the lower chambers of six-well plates and cultured in a medium containing 100 μM H_2_O_2_ for 5 h to induce the formation of senescence. After exposure to 100 μM H_2_O_2_ for 5 h, the KGN cells were cocultured with BMSCs in the transwell system. Approximately 5 × 10^5^ BMSCs were seeded into the upper chambers of six-well hanging cell culture inserts (Merck Millipore, Darmstadt, Germany). Then the KGN cells were cocultured with BMSCs for 48 h, the KGN cells were collected for further study. KGN cells were planted into 6-well plates one day before treatment. When the cells grew at about 70% of confluent, 2 μg of exosomes were directly added into the cells. PBS was added as a control. After 48 h, cells were collected for further experiments.

### In Vivo Ovarian Premature Failure Model

All animal experiment protocols were approved by the Institutional Animal Care and Use Committee (IACUC) of the Medical School of Jiangsu University, according to the laboratory guidelines for the ethical review of animal welfare. Female clean-grade Sprague Dawley (SD) rats were purchased from Jiangsu University Experimental Animal Center. Five-week-old female SD rats with regular estrous cycles were intraperitoneally injected with CTX (50 mg/kg) on the first day and then consecutive injected for 13 days (8 mg/kg/d). Vaginal smears and weight were obtained to assess the modeling effect from the first day of injection. After successful modeling, all modeled rats were randomly assigned into four groups and were injected with PBS (100 μL), BMSCs-Exos (150 μg/100 μL PBS), Exos-si-circLRRC8A (150 μg/100 μL PBS), and Exos-oe-circLRRC8A (150 μg/100 μL PBS) by tail vein respectively every two days.

### Elisa

The serum hormone concentrations, including AMH, FSH, LH, and E2 were monitored by enzyme-linked immunosorbent assay kits (ImmunoWay, USA). The serum samples were collected and incubated with ninety-six-well plates with antibodies at room temperature for 2 h. The serum hormone levels were measured by using a microplate reader.

### H&E and Immunohistochemistry

The rat ovaries were fixed in 4% paraformaldehyde solution, embedded in paraffin, and cut into 4 μm thick sections. The sections were stained with hematoxylin and eosin (H&E) for routine histological examination. For immunohistochemical analyses, the sections were incubated with primary antibody against NFE2L1 (Proteintech,17,062-1-AP,1:100). After being incubated at 4 °C for one night, the sections were incubated with the HRP-polymer-conjugated secondary antibody at room temperature for 1 h. These samples were then stained with a 3,3-diaminobenzidine solution (DAB) for 10 mins to visualize the image. Finally, the sections were then stained with hematoxylins for 1 min. The images were captured on Leica fluorescence microscope.

### Statistical Analysis

All data were based on three independent experiments and were presented as the mean ± SD. Student’s t test was used to assess the statistical significance between the two groups. Differences between two or more groups were assessed using one-way ANOVA. Statistical analyses were performed using GraphPad Prism 9 (GraphPad Software, USA). *P*-values <0.05 was considered statistically significant (*P < 0.05; **P < 0.01; ***P < 0.001, ****P < 0.0001) in this study.

## Results

### BMSCs-Derived Exosomes Alleviate Oxidative Damage of GCs

**Fig. 1:**
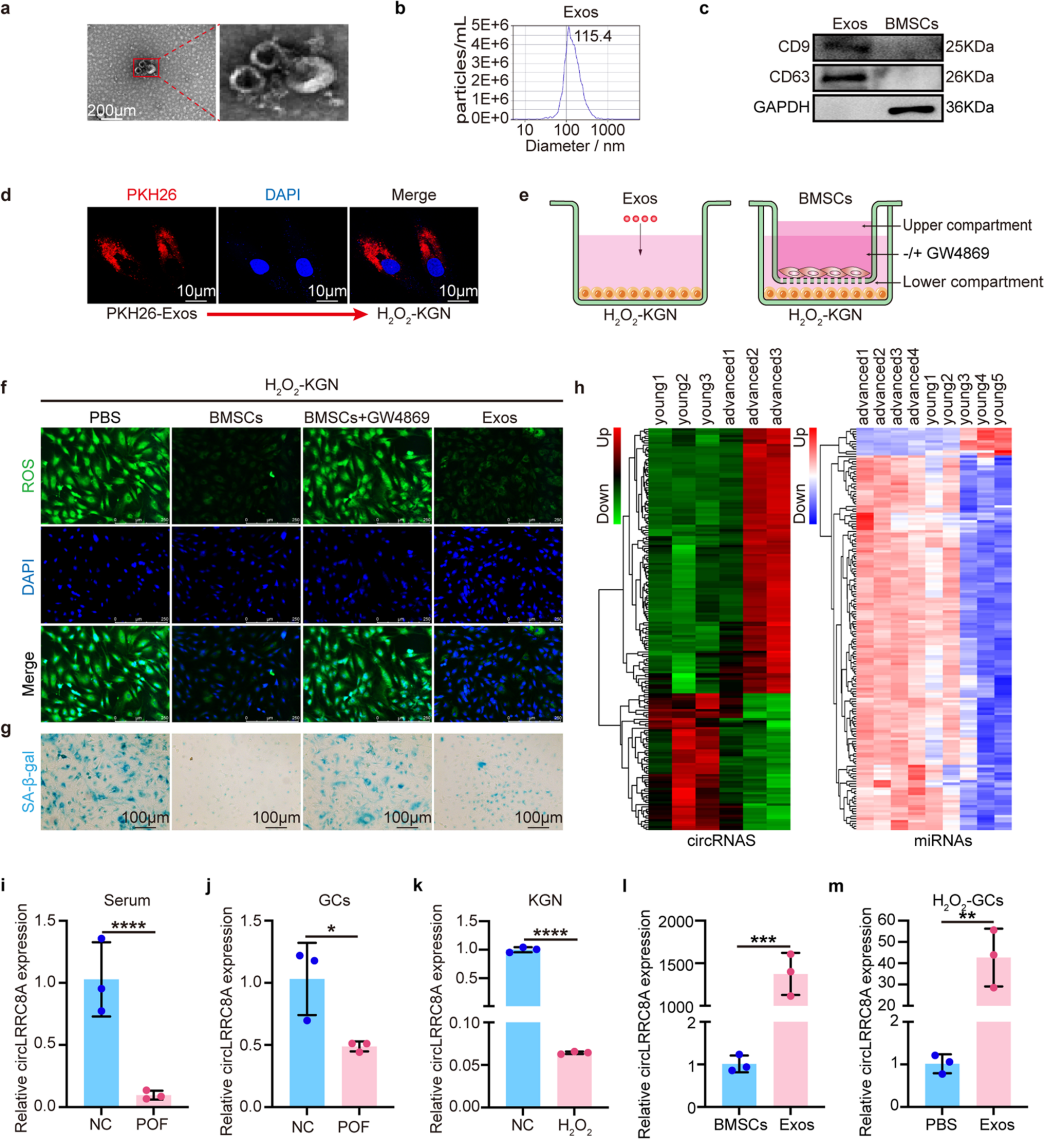
CircLRRC8A is significantly upregulated in oxidatively damaged GCs treated with BMSCs-derived exosomes. **a** Image of Exos observed by transmission electron microscope. **b** Nanoparticle tracking analysis of size distribution and concentration of Exos. **c** Western blot analysis of exosome-related markers CD9 and CD63. **d** KGN cells were incubated with PKH26-labeled Exos for 24 h and Exos uptake was detected by fluorescence microscopy. Red: PKH26-Exos staining; Blue: nuclear staining. **e** Schematic diagrams of Exos/BMSCs and H_2_O_2_-KGN cells co-culture system. **f** The reactive oxygen species levels were detected by DCFH-DA staining in the PBS, BMSCs, BMSCs+GW4869, and Exos groups. Green: DCFH-DA staining; Blue: nuclear staining. **g** SA-β-gal staining. Senescent cells were stained blue. The ratio of SA-β-gal-positive cells was much higher in PBS and BMSCs+GW4869 groups than BMSCs and Exos groups. **h** Hierarchical clustering of differentially expressed circRNAs (left) and miRNAs (right) in samples of granulosa cells from women with advanced age (AA, ≥ 38 years) and young age (YA, ≤ 30 years). **i-l** Relative expression of circLRRC8A in the serum sample, clinical sample, senescent model, and Exos were determined by qRT-PCR (*n* = 3). **m** The expression of the circLRRC8A was verified in H_2_O_2_-GCs with or without Exos treatment (*n* = 3)

Our previous studies revealed that BMSCs-Exos are linked to the functional repair of ovarian aging [16]. To explore the underlying mechanism, we first evaluated the characteristics of purified nanoparticles derived from BMSCs. Transmission electron microscopy (TEM) exhibited a typical cup-shaped structure and a size of approximately 100 nm (Fig. 1a). Nanoparticle tracking analysis (NTA) showed that the diameter was 115.4 nm (Fig. 1b). The exosomal surface markers CD9 and CD63 were highly expressed in Exos (Fig. 1c). As shown, PKH26-labeled Exos were absorbed by KGN cells (Fig. 1d). To evaluate the therapeutic effect of Exos on oxidative injury in GCs, H_2_O_2_ pretreated KGN cells were incubated with BMSCs with or without 10 μM GW4869 (Sigma Aldrich) (inhibitors of exosome secretion) for 48 h, as well as Exos (Fig. 1e). ROS assays showed that Exos significantly reduced KGN cells oxidative damage relative to that in the control group (Fig. 1f and Supplementary Fig. 1d). SA-β-gal staining assays (a widely used senescence marker) showed that Exos markedly decreased KGN cellular senescence (Fig. 1g and Supplementary Fig. 1d). These results indicate that BMSCs-Exos have the potential to inhibit granulosa cells senescence induced by H_2_O_2_.

### CircLRRC8A Is Significantly Upregulated in Oxidatively Damaged GCs Treated with BMSCs-Derived Exosomes

Exosomes contain functional miRNAs and circRNAs. MiRNAs play an important role in restoring ovarian function [16, 27-29], however, the role of circRNAs in ovarian repair has not been explored. We downloaded the circRNA expression profile of human granulosa cells during maternal aging with the accession number GSE97193 from the GEO database, which contained 3 women with advanced age (AA, ≥ 38 years) from IVF patients and 3 matched women with young age (YA, ≤ 30 years) from IVF patients. Human follicular fluid miRNA expression matrices of 5 advanced age (AA, >36 years old) patients and 5 matched young age (YA, < 36 years old) were downloaded from the GSE63737 dataset for miRNA analysis. We analyzed online datasets of circRNAs and miRNA profiles (GSE97193 and GSE63737) in GCs and follicular fluid samples of females of different ages. Bioinformatics analysis suggested that the expression of circRNAs and miRNAs varied between young and advanced-aged females. Results of differential circRNA analysis are shown in the heatmap and the volcano plot (Fig. 1h and Supplementary Fig. 1e), filtered by *P* < 0.05 and log2FC > 2 to obtain 118 were upregulated and 61 were downregulated circRNAs. Results of miRNA differential analysis are shown in the heatmap (Fig. 1h), a total of 174 differential expressions miRNAs were obtained by filtering at *P* < 0.05 and log2FC > 0.5. Based on upregulated miRNAs, target circRNAs were predicted using the miRanda and PITA databases. The predicted circRNAs were intersected with the above downregulated circRNAs to obtain 25 differential circRNAs and their corresponding target miRNAs. We built competing endogenous RNA networks between the downregulated circRNAs and the corresponding upregulated miRNAs (Supplementary Fig. 1f). When compared with all other circRNA candidates in circRNAs-miRNAs networks, circLRRC8A was found to be one of the most downregulated circRNAs in GCs from human follicular fluids treated with hydrogen peroxide (Supplementary Fig. 1g). We further detected its expression in serum samples and GCs, including proliferating KGN cells, primary human GCs derived from patients with normal ovarian reserve undergoing in vitro fertilization and senescent cells (KGN cells treated with hydrogen peroxide and primary human GCs derived from POF patients), and found that circLRRC8A was obviously reduced in senescent cells (Fig. 1i-k). Interestingly, the content of circLRRC8A in Exos was significantly higher than that in parental BMSCs (Fig. 1l). To further validate the association of circLRRC8A with Exos-mediated therapeutic effects on oxidatively damaged GCs, we detected its expression in GCs pretreated with H_2_O_2_ with or without Exos addition and found that circLRRC8A showed much higher levels in GCs after Exos addition (Fig. 1m), implying that circLRRC8A may be related to GCs oxidative damage repair mediated by Exos. Thus, our subsequent studies focused on whether circLRRC8A could regulate GCs senescence due to oxidative damage and the potential mechanisms underlying the benefits of circLRRC8A-enriched Exos-based therapies.

### Identifications and Characteristics of circLRRC8A in GCs

**Fig. 2:**
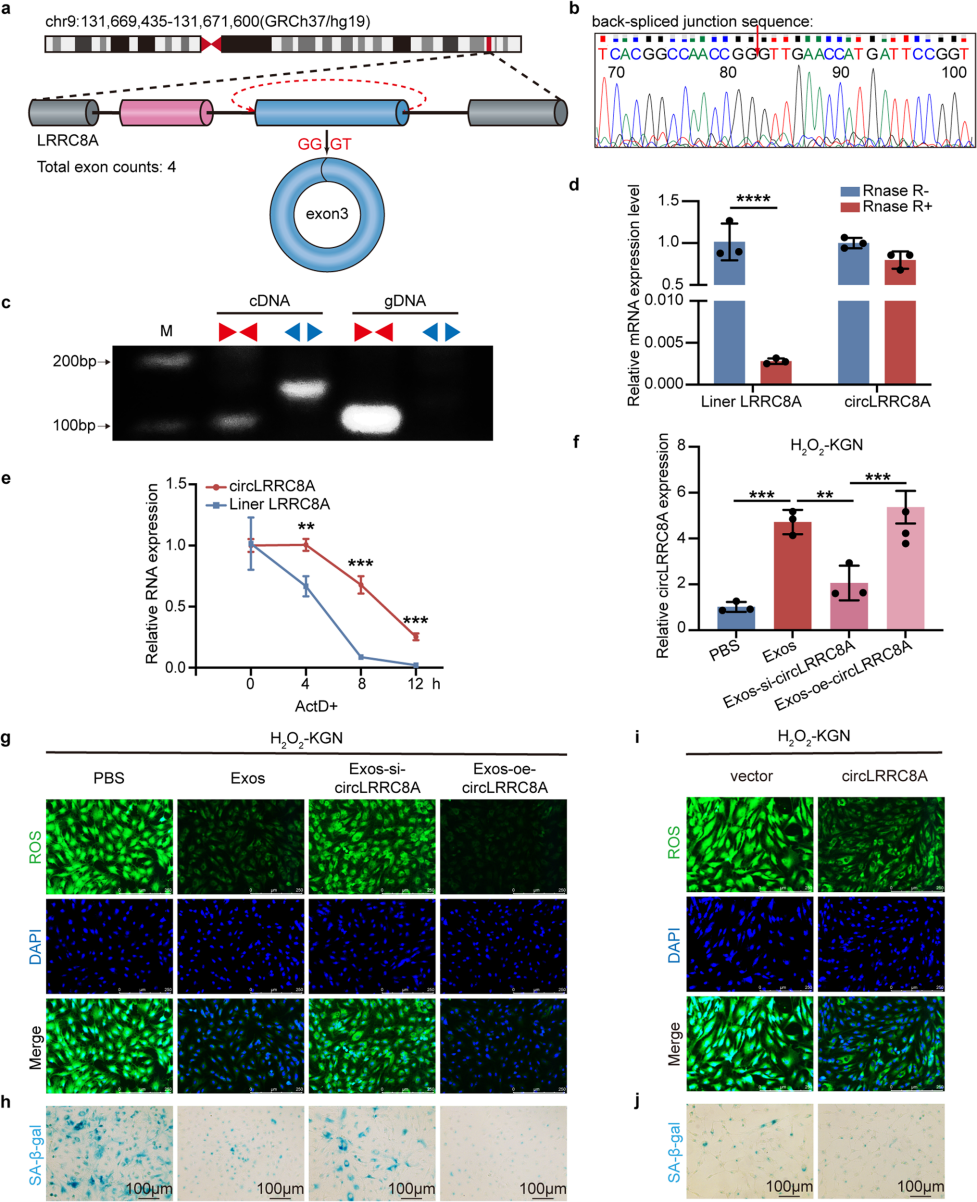
BMSCs-Exos rescued oxidative damage in KGN cells by releasing circLRRC8A. **a** Schematic diagram of the genomic location and splicing pattern of hsa_circ_0007311 (2165 bp). **b** The arrow represents the “head to tail” splicing sites of circLRRC8A identified by Sanger sequencing. **c** The expression levels of the back spliced and canonical forms of LRRC8A in cDNA and gDNA isolated from KGN cells. Red arrows represent convergent primers; blue arrows represent divergent primers. **d** Relative expression of circLRRC8A and linear LRRC8A in the presence or absence of RNase R supplementation (*n* = 3). **e** The abundances of circLRRC8A and linear LRRC8A in KGN cells treated with actinomycin D at the indicated time points (*n* = 3). **f** qRT-PCR analysis of circLRRC8A expression in H_2_O_2_-KGN cells that were treated with PBS, Exos, Exos-si-circLRRC8A or Exos-oe-circLRRC8A (*n* = 3). **g, h** The ROS staining and SA-β-gal staining demonstrated that oxidative damage and senescence of KGN cells were reduced when cocultured with exosomes secreted from BMSCs transfected with siRNA or overexpression plasmid of circLRRC8A. **i, j** ROS levels and SA-β-gal staining assays in H_2_O_2_-KGN cells transfected by mock plasmids and circLRRC8A plasmids

To validate the existence of circLRRC8A, we matched hsa_circ_0007311 (termed circLRRC8A throughout the rest of the article) with data in NCBI (https://www.ncbi.nlm.nih.gov/) and found that circLRRC8A was generated from exon 3 of the LRRC8A gene via back-splicing, with a transcript of 2165 nucleotides (Fig. 2a). Convergent primers across the junction site were designed, and the reverse transcription product was confirmed by Sanger sequencing (Fig. 2b). Agarose gel electrophoresis of qRT-PCR products showed that divergent primers could yield a specific band in cDNA but not gDNA (Fig. 2c). Furthermore, circLRRC8A could endure RNase R digestion, while the linear form product was adverse (Fig. 2d). Then, we detected the stability of circLRRC8A by treating KGN cells with actinomycin D (ActD, a transcription inhibitor), and the results confirmed that the transcriptional half-life of linear LRRC8A was shorter than that of its circular transcript (Fig. 2e). These results suggest that circLRRC8A can be an ideal molecules ideal candidate for disease.

### BMSCs-Exos Rescued Oxidative Damage in KGN Cells by Releasing circLRRC8A

Considering the abundance of circLRRC8A in Exos, we hypothesized that circLRRC8A may play an important role in the therapeutic effect of Exos on oxidatively damaged KGN cells. To clarify this, we first considered the possibility that the linear LRRC8A may also play a role in premature ovarian failure. Therefore, we examined the expression of linear LRRC8A in exosomes and showed that linear LRRC8A was absent in Exos (Supplementary Fig. 2a). Furthermore, we verified the knockdown specificity of Exo-si-circLRRC8A for circLRRC8A, Exos-si-circLRRC8 significantly reduced the expression of circLRRC8A in H_2_O_2_-KGN, but not target the linear form (Supplementary Fig. 2b-c). H_2_O_2_-pretreated KGN cells were cocultured with exosomes secreted from BMSCs transfected with siRNA (Exos-si-circLRRC8A) or the overexpression plasmid circLRRC8A (Exos-oe-circLRRC8A) (Fig. 2f). Importantly, compared with Exos, Exos-si-circLRRC8A contributed to increased ROS levels and SA-β gal activity, revealing increased oxidative injury and senescence in KGN cells (Fig. 2g-h and Supplementary Fig. 2d-e), while Exos-oe-circLRRC8A addition exhibited a stronger therapeutic effect on senescent KGN cells. Collectively, circLRRC8A may be a key molecule by which Exos reduces cellular senescence caused by oxidative damage. Next, the role of circLRRC8A in GCs senescence was investigated. As shown in Fig. 2i-j and Supplementary Fig. 2f-h, directly elevated circLRRC8A in GCs led to reduced ROS levels and decreased SA-β gal activity accompanied by decreased levels of the senescence marker P21 (Supplementary Fig. 2i-j). Together, our results illustrate that circLRRC8A could prevent GCs senescence triggered by oxidative damage.

### CircLRRC8A Serves as a Sponge of miR-125a-3p

**Fig. 3:**
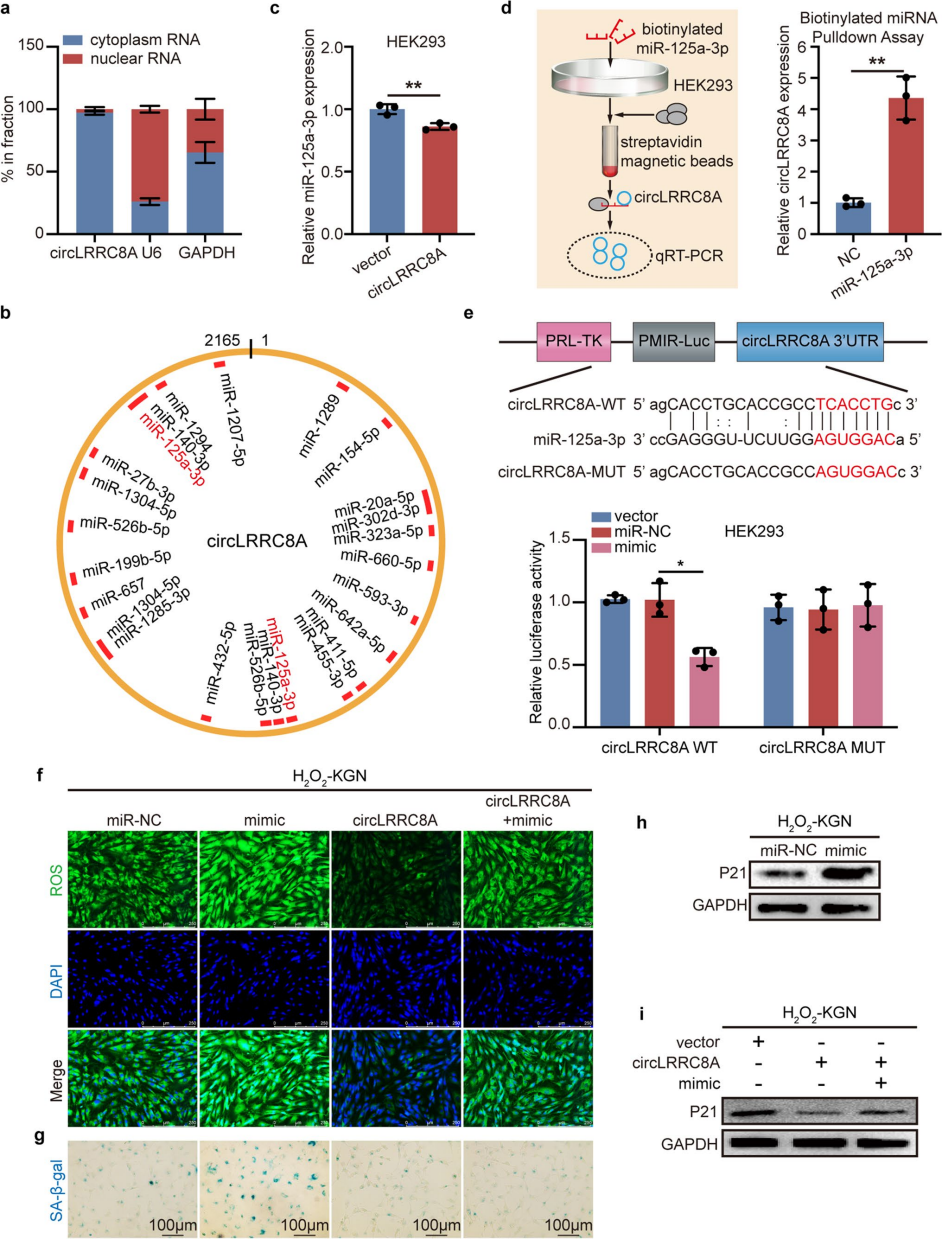
CircLRRC8A serves as a sponge of miR-125a-3p. **a** Relative circLRRC8A expression levels in nuclear and cytosolic fractions of KGN cells are shown (*n* = 3). U6 was used as the nuclear control. GAPDH was used as the cytosolic control. **b** A schematic drawing shows the putative binding sites for miRNAs associated with circLRRC8A. **c** The expression of the miR-125a-3p in HEK293 cells transfected with circLRRC8A plasmid (*n* = 3). **d** RT-PCR analysis of circLRRC8A in the pull-down lysates of biotinylated miR-125a-3p (*n* = 3). **e** A luciferase reporter assay was used to evaluate the luciferase activity of p-luc-circLRRC8A in HEK293 cells transfected with miR-125a-3p mimics to identify miR-125a-3p that were able to bind to the circLRRC8A (*n* = 3). **f** Assessment of the oxidative damage of KGN cells transfected with miR-NC, miR-125a-3p, circLRRC8A plasmid, or cotransfected with miR-125a-3p mimic and circLRRC8A plasmid by ROS staining. **g** Assessment of the senescence of KGN cells transfected with miR-NC, miR-125a-3p, circLRRC8A plasmid, or cotransfected with miR-125a-3p mimic and circLRRC8A plasmid by SA-β-gal staining. **h** The relative protein levels of P21 in H_2_O_2_-KGN cells transfected with miR-NC and miR-125a-3p mimics were determined. **i** Relative protein levels of P21 in H_2_O_2_-KGN cells transfected with vector plasmid, circLRRC8A plasmid, or circLRRC8A plasmid along with miR-125a-3p mimic

Given the above evidence, we investigated how circLRRC8A regulates the cellular senescence phenotype. CircRNAs have been reported to be able to enhance or suppress gene expression by modulating the availability of microRNAs for target mRNAs. The nuclear mass separation assays revealed that circLRRC8A was mainly localized in the cytoplasm of KGN cells (Fig. 3a). Combined with the high stability of circLRRC8A, we hypothesized that circLRRC8A may potentially act as a ‘sponge’ or a ‘decoy’, inhibiting the activity of associated microRNAs. Twenty-one candidate miRNAs were predicted to have binding sites along the circLRRC8A sequences using the miRanda database (Fig. 3b). Among these predicted miRNAs, miR-1207-5p, miR-125a-3p, miR-1289, miR-1294, miR-1304-5p, miR-323a-5p, miR-432-5p, and miR-657 were found to be associated with circLRRC8A in PITA database. To verify the vital miRNAs that may interact with circLRRC8A, we transfected a circLRRC8A overexpression plasmid into HEK293 cells and detected the 8 predicted miRNAs which in the circRNAs-miRNAs networks using qRT-PCR. We found that circLRRC8A overexpression significantly reduced miR-125a-3p in HEK293 cells (Fig. 3c), and the specificity for the pulldown of circLRRC8A by the biotinylated miR-125a-3p probe was confirmed by qRT-PCR analysis (Fig. 3d). To further measure the binding between circLRRC8A and miR-125a-3p, dual-luciferase reporter assays were performed. The wild-type and mutant dual-luciferase reporter plasmids of circLRRC8A were constructed, and the data indicated that the miR-125a-3p mimic obviously reduced the luciferase activity of the circLRRRC8A-WT luciferase reporter but not that of the mutants (Fig. 3e). All these results proved that circLRRC8A serves as a sponge of miR-125a-3p. Not surprisingly, miR-125a-3p was upregulated in senescent GCs (Supplementary Fig. 3a). To confirm the biological functions of miR-125a-3p and whether circLRRC8A affects the function of miR-125a-3p, rescue assays were performed. ROS and SA-β-gal staining assays showed that the miR-125a-3p mimic aggravated KGN cells oxidative injury and senescence, but circLRRC8A overexpression reversed this aggravation of GCs development (Fig. 3f-g and Supplementary Fig. 3b). Consistently, we confirmed that the protein levels of P21 were increased by miR-125a-3p mimics, whereas cotransfection of oe-circLRRC8A and miR-125a-3p mimics reversed this effect (Fig. 3h-i and Supplementary Fig. 3c-d). Together, the above data reveal that circLRRC8A regulates GCs senescence caused by oxidative damage by inhibiting miR-125a-3p.

### CircLRRC8A Upregulates NFE2L1 Expression by Sponging miR-125a-3p

**Fig. 4:**
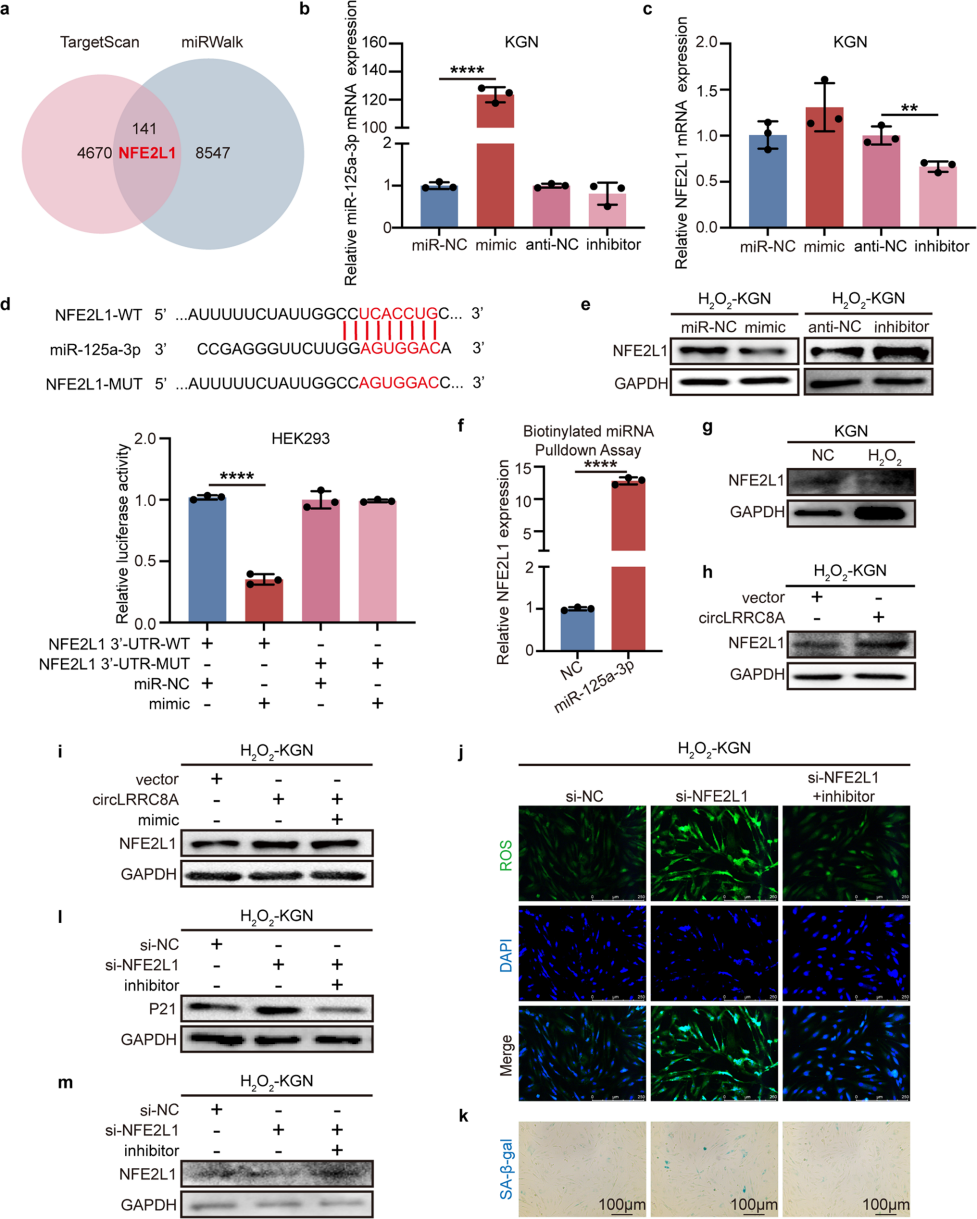
CircLRRC8A upregulates NFE2L1 expression by sponging miR-125a-3p. **a** A schematic drawing of the screening procedure for miR-125a-3p candidate targets. **b, c** The relative mRNA levels in H_2_O_2_-KGN cells transfected with miR-NC and miR-125a-3p mimics were determined (*n* = 3). **d** A luciferase reporter plasmid carrying wildtype (WT) or mutant (MUT) NFE2L1 were cotransfected into HEK293 cells with miR-125a-3p mimics in parallel with an empty vector. Relative luciferase activity in the HEK293 cells was determined (*n* = 3). **e** The relative protein levels in H_2_O_2_-KGN cells transfected with miR-NC, miR-125a-3p mimics, inhibitor-NC, and inhibitor were determined. **f** qRT-PCR analysis of pull-down by biotinylated miR-125a-3p (*n* = 3). **g** Relative protein levels of NFE2L1 in H_2_O_2_-KGN cells. **h** The western blot showed that circLRRC8A overexpression resulted in increased expression of NFE2L1 in H_2_O_2_-KGN cells. **i** Relative protein levels of NFE2L1 in H_2_O_2_-KGN cells transfected with vector plasmid, circLRRC8A plasmid, or circLRRC8A plasmid along with miR-125a-3p mimics. **j, k** ROS staining, and SA-β-gal staining revealing the effect of si-NFE2L1 on senescent-KGN cells after knocking down miR-125a-3p expression. **l, m** Western blot analysis of the expression of P21 and NFE2L1 after cotransfection of a miR-125a-3p inhibitor and si-NEF2L1 into senescent-KGN cells

The target gene of miR-125a-3p was predicted by using bioinformatics analysis websites (TargetScan and miRWalk). Given the implication of NFE2L1 in oxidative stress regulation and cell senescence, we finally selected NFE2L1 from 141 putative target protein candidates as a potential signaling pathway downstream of circLRRC8A/miR-125a-3p (Fig. 4a). Upregulation of miR-125a-3p decreased NFE2L1 expression, while anti-miR-125a-3p exhibited the opposite effect (Fig. 4b-c). Furthermore, miR-125a-3p directly bound to the NFE2L1 3′ untranslated region (UTR) (Fig. 4d). NFE2L1 protein levels were increased and decreased by miR-125a-3p inhibitor and mimic, respectively (Fig. 4e and Supplementary Fig. 4a). A biotinylated miR-125a-3p pulldown assay showed significant enrichment of NFE2L1 compared with the negative control in KGN cells (Fig. 4f). Consistently, we confirmed that the expression of NFE2L1 had the same tendency as circLRRC8A (Fig. 4g and Supplementary Fig. 4b). Moreover, oe-circLRRC8A increased NFE2L1 mRNA and protein levels (Fig. 4h and Supplementary Fig. 4c). However, the increased NFE2L1 protein levels were decreased by the miR-125a-3p mimic (Fig. 4i and Supplementary Fig. 4d). We observed that knocking down NFE2L1 expression aggravated oxidative injury and senescence in H_2_O_2_-KGN cells, while inhibition of miR-125a-3p restored the deterioration in NFE2L1-deleted senescent KGN cells (Fig. 4j-k and Supplementary Fig. 4f-g). Si-NFE2L1 decreased the P21 protein levels, while weakening miR-125a-3p reversed this effect (Fig. 4l and Supplementary Fig.4 h). Furthermore, an inhibitor of miR-125a-3p activated NFE2L1 expression (Fig. 4m and Supplementary Fig. 4i). Thus, these results demonstrate that circLRRC8A induces NFE2L1 expression by functioning as a negative regulator of miR-125a-3p and thus suppresses GCs senescence.

### RNA Binding Protein EIF4A3 Facilitates the Biogenesis of circLRRC8A

**Fig. 5:**
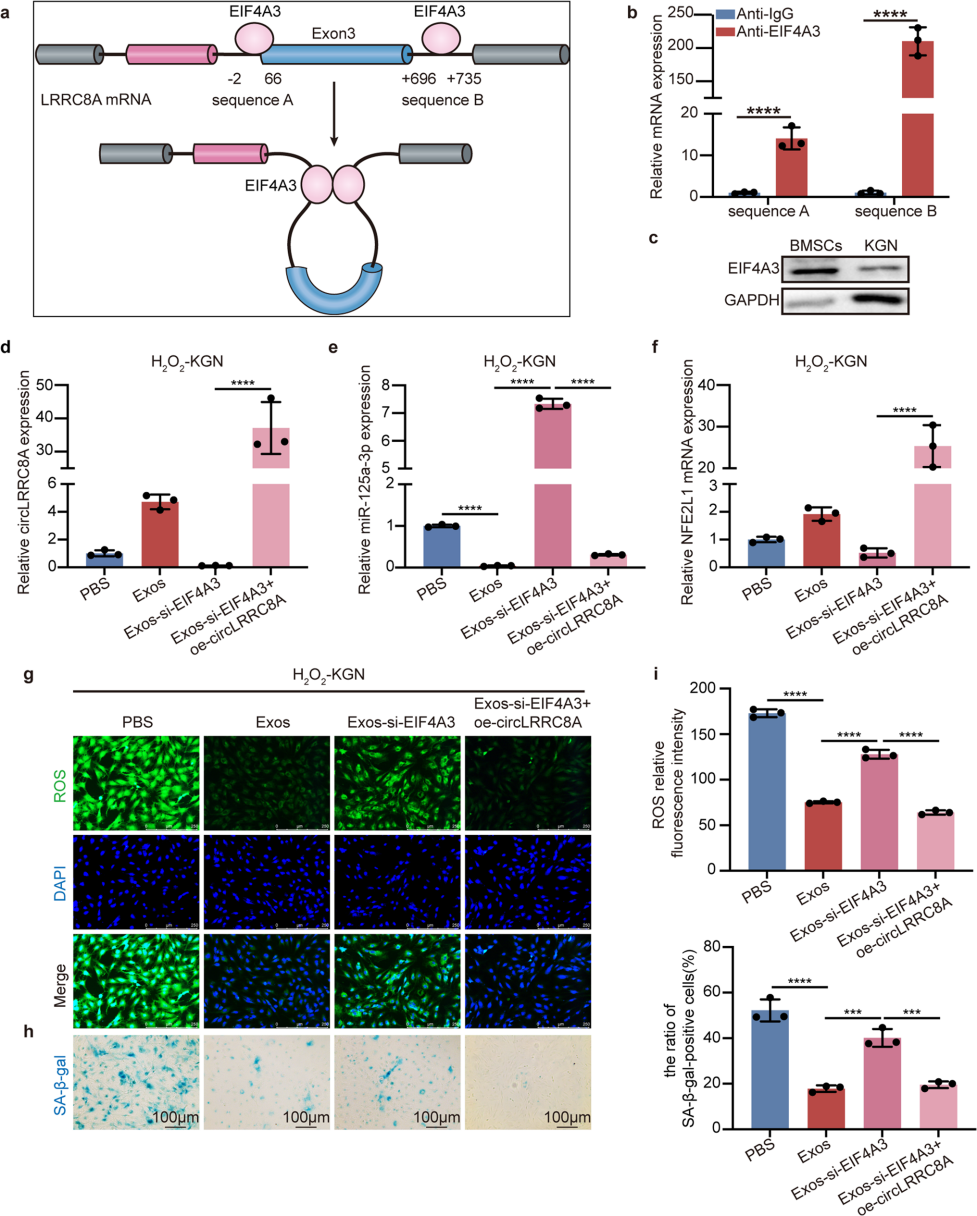
RNA Binding Protein EIF4A3 facilitates the biogenesis of circLRRC8A. **a** The binding sites for EIF4A3 in the flanking sequences of the LRRC8A mRNA transcript were predicted using CircInteractome. **b** RIP assays to verify EIF4A3 binding at the putative sites (*n* = 3). **c** The protein levels of EIF4A3 expression in BMSCs and KGN cells. **d-f** qRT-PCR analysis of expression of circLRRC8A, miR-125a-3p, and NFE2L1 in H_2_O_2_-KGN cells that were treated with PBS, Exos, Exos-si-EIF4A3, and Exos-si-EIF4A3 + circLRRC8A (*n* = 3). **g-i** ROS staining and SA-β-gal staining in senescent-KGN cells cocultured with PBS, Exos, Exos-si-EIF4A3, and Exos-si-EIF4A3 + circLRRC8A (*n* = 3)

RNA-binding proteins can mediate circRNA cyclization by targeting specific motifs in the flanking intron [30]. Two binding sites of EIF4A3 were predicted in the upstream and downstream regions of circLRRC8A, called sequence A and sequence B in the CircInteractome database (https://circinteractome.irp.nia.nih.gov/) (Fig. 5a). The RNA immunoprecipitation (RIP) experiment confirmed that EIF4A3 was able to bind with two predicted binding sites of LRRC8A pre-mRNA, especially sequence B (Fig. 5b). We detected higher EIF4A3 expression levels in BMSCs than in KGN cells, and EIF4A3 was reduced upon H_2_O_2_ treatment in KGN cells (Fig. 5c and Supplementary Fig. 5a-c). In addition, EIF4A3 silencing in BMSCs induced miR-125a-3p expression, accompanied by a significant reduction in circLRRC8A and NFE2L1 (Supplementary Fig. 5d-h). To further confirm that the observed circLRRC8A-mediated phenotypes could be regulated by the dysregulation of EIF4A3, we performed functional rescue assays. As shown in Fig. 5d-i, after coculture with Exos derived from the culture medium of EIFEA3 siRNA-transfected BMSCs (Exos-si-EIF4A3), circLRRC8A and NFE2L1 were significantly downregulated, accompanied by increased ROS and cellular senescence in GCs and upregulated miR-125a-3p, which could be reversed by circLRRC8A overexpression in KGN cells. Our data suggest that circLRRC8A could be cyclized by EIF4A3 and that this process is involved in BMSCs-Exos-based oxidative damage repair in GCs.

### Exos-circLRRC8A Regulates GCs Senescence in POF In Vivo

**Fig. 6:**
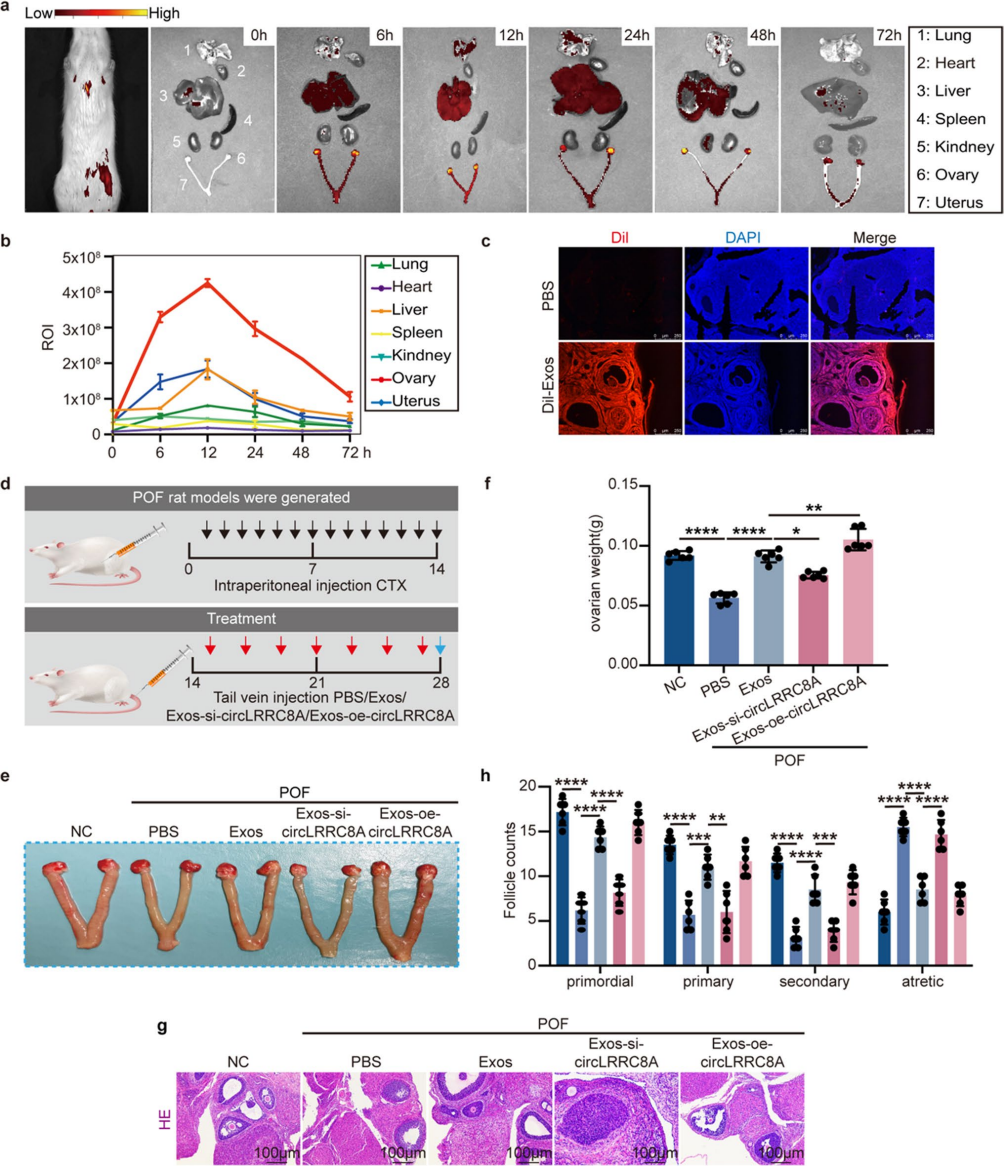
Exos-circLRRC8A regulates GCs senescence in POF in vivo. **a** Fluorescent images of major organs (1) lung, (2) heart, (3) liver, (4) spleen, (5) kidney, (6) ovary, (7) uterus at 0, 6, 12, 24, 48, and 72 h post-injection of Exos. Red: low fluorescence intensity; orange: moderate fluorescence intensity; yellow: high fluorescence intensity. **b** Statistical diagram of fluorescence intensity in organs. **c** Fluorescence localization in ovarian frozen sections (*n* = 3). red: DiI; blue: DAPI. **d** Flow Chart of rat experiment (*n* = 6). **e** Representative ovaries at 14 days after transplantation. **f** Ovarian weight (*n* = 6). **g** H&E staining of ovarian sections. **h** Counting of follicles at different stages (*n* = 6)

To elucidate whether Exos-circLRRC8A could repair oxidatively damaged ovarian function in vivo, we first explored the biodistribution of Exos. Rats were intravenously injected with Dil-labeled Exos (Dil-Exos) (100 μg) and then imaged using an in vivo imaging system (IVIS) at different times postinjection. Analysis of the organs excised 0, 6, 12, 24, 48, and 72 h post-injection showed accumulation of Dil-Exos mainly in the ovary, uterus, liver, lung, and kidney (Fig. 6a). The fluorescence intensity in each organ changed with time and was strongest at 12 h (Fig. 6b). Importantly, we detected obvious Dil signals in frozen ovarian sections, especially in the ovarian cortex (Fig. 6c). Next, POF rat models were generated by intraperitoneal injection CTX as previously described [16]. Two weeks after CTX injection, rats were injected via the tail vein with PBS, Exos, Exos-si-circLRRC8A, or Exos-oe-circLRRC8A (Fig. 6d). The results illustrated that ovaries derived from Exos and Exos-oe-circLRRC8A were significantly larger than those from the Exos-si-circLRRC8A group, in terms of size, weight, and ovarian ratio (Fig. 6e-f and Supplementary Fig. 6a), suggesting that circLRRC8A knockdown limited ovarian growth. We analyzed the stages of the estrous cycle in each group, and Exos or Exos-oe-circLRRC8A treatment restored the disturbance of the estrous cycle caused by CTX (Supplementary Fig. 6b-c). Furthermore, we detected the serum levels of E_2_, AMH, LH, and FSH in each group. Compared with PBS treatment, the serum concentration of AMH and E_2_ was enhanced in POF rats with Exos or Exos-oe-circLRRC8A treatment, while FSH and LH levels were reduced. However, there was no difference in the levels of these hormones between the Exos group and the Exos-oe-circLRRC8A group (Supplementary Fig. 6d). Histopathological examination showed that there were several atresia follicles in the PBS group. Exos and Exos-oe-circLRRC8A transplantation restored the reduction in functional follicles and the increase in closed follicles originating from CTX treatment. In contrast, functional follicles were absent in the Exos-si-circLRRC8A group (Fig. 6g-h). To detect whether Exos exerted harmful effects on other organs, histopathology was implemented. There was no significant pathological damage to the heart, liver, spleen, lung, or kidney in the Exos group (Supplementary Fig. 6e). The results showed that Exos caused no obvious pathological damage to other organs and may become a promising treatment strategy for POF.

### Exos-circLRRC8A Regulate GCs Senescence Via the miR-125a-3p/NFE2L1 Signaling Pathway In Vivo

**Fig. 7:**
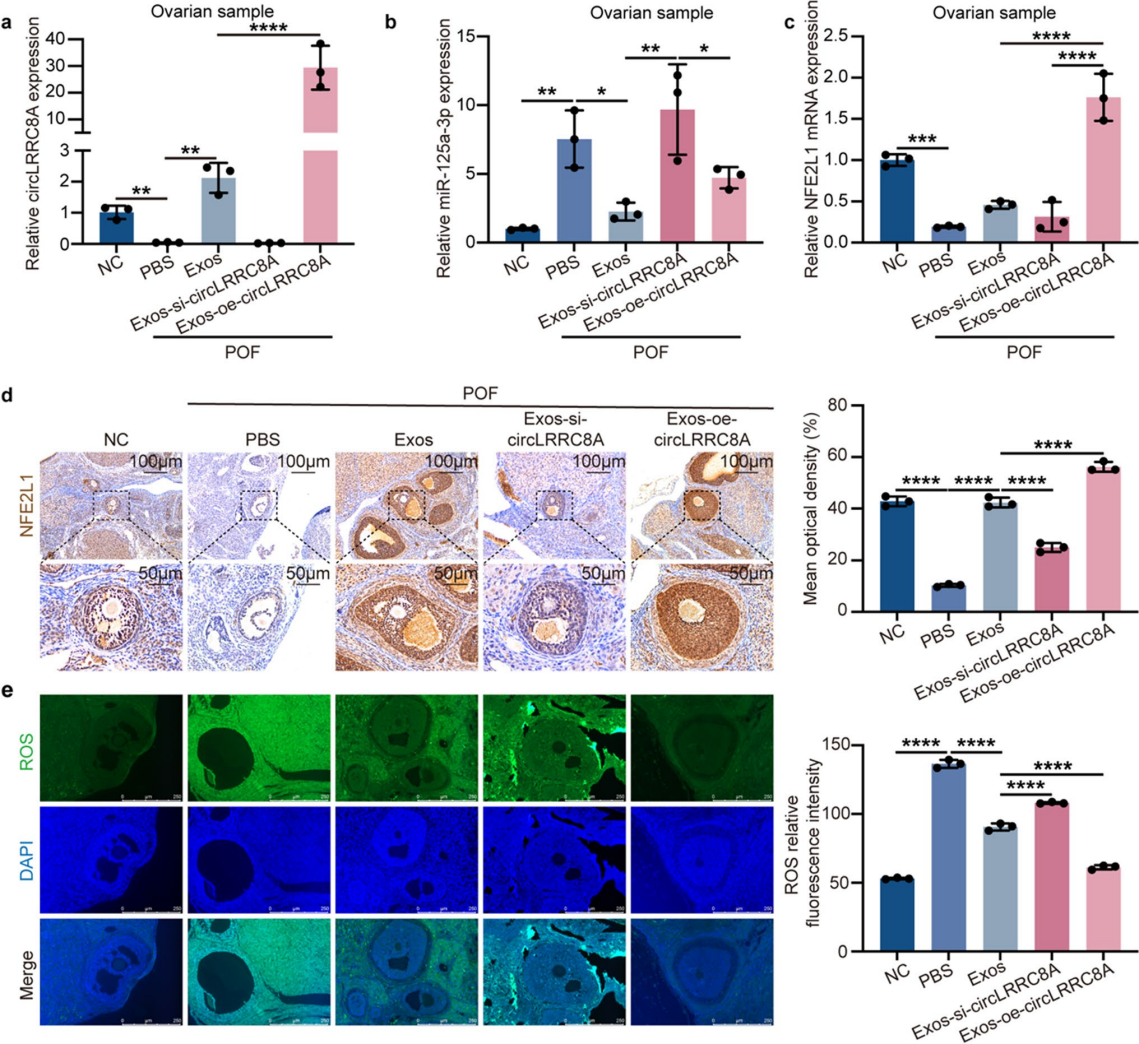
Exos-circLRRC8A regulates GCs senescence by miR-125a-3p/NFE2L1 signaling pathway in vivo. **a-c** qRT-PCR analysis of expression of circLRRC8A, miR-125a-3p, and NFE2L1 in rat ovarian tissue (*n* = 3). **d** IHC staining of NFE2L1 in rat ovarian tissue (*n* = 3). **e** ROS levels detection of ovarian sections (*n* = 3)

**Fig. 8:**
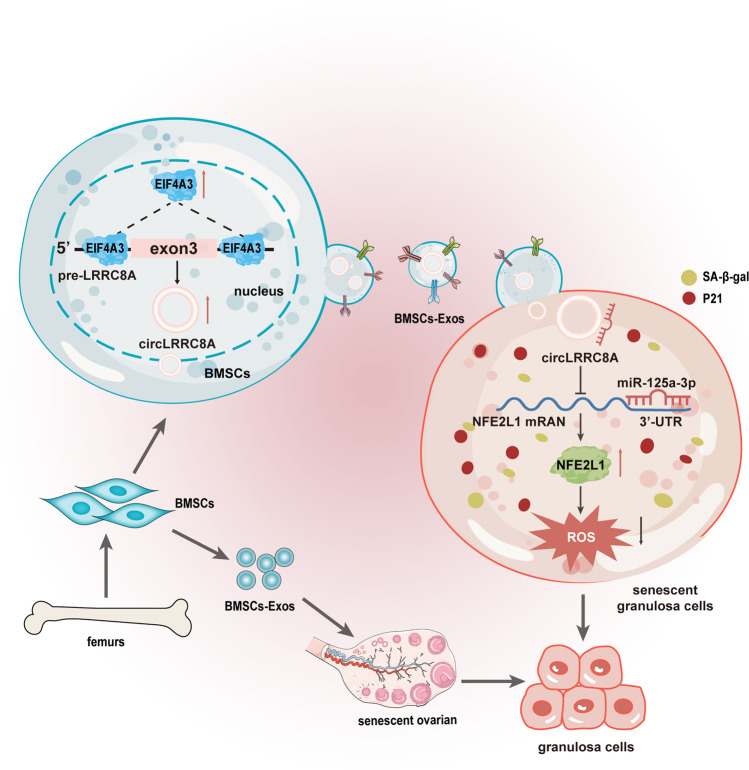
Schematic of the mechanisms involving exosomal circLRRC8A in the regulation of POF

Next, we extracted total RNA from rat ovarian tissue to investigate circLRRC8A by qRT-PCR. The results demonstrated that the circLRRC8A levels in ovarian tissue were dependent on exosomal circLRRC8A expression (Fig. 7a), and miR-125a-3p was negatively correlated with circLRRC8A (Fig. 7b). Moreover, NFE2L1 mRNA and protein levels were decreased in the Exos-si-circLRRC8A group and increased in the Exos and Exos-oe-circLRRC8A group compared to the control group (Fig. 7c and d). Undoubtedly, Exos and Exos-oe-circLRRC8A treatment effectively reduced the oxidative injury in POF compared to that in the PBS group. A depressor effect was not found in the Exos-si-circLRRC8A treatment group (Fig. 7e). All these results confirmed that circLRRC8A could be delivered via Exos to alleviate oxidative damage in vivo by regulating the miR-125a-3p/NFE2L1 axis (Fig. 8).

## Discussion

POF not only leads to a decline in female reproductive endocrine function but also affects patients’ psychological condition and the function of other organs [31]. A widely accepted concept is that oxidative damage results in compromised follicles in POF linked to fecundity decreases [32, 33]. Our previous studies have affirmed the therapeutic effects of BMCs-derived exosomes on ovarian function in a cell-free therapeutic manner [16]; however, the key performers in the BMSCs-Exos-mediated repair process and the underlying mechanisms need to be further addressed. Here, we found that in senescent GCs, MSCs extracellular vesicles (MSCs-EVs) treatment protected against senescence-induced biomarkers and dysfunctions and improved ROS levels.

We have characterized a new function for a circular RNA derived from the LRRC8A gene, circLRRC8A, on oxidative damage-induced cellular senescence. The decrease in circLRRC8A levels in the POF model (in vivo and *vitro*) and in GCs derived from human follicular fluid appeared to be functionally important because knockdown of circLRRC8A alone promoted GCs senescence. We further elucidated the mechanism whereby circLRRC8A elicited this effect and found a potential function for circLRRC8A as an inhibitor or sponge of miR-125a-3p. Then, the oxidative damage-triggered senescence repaired by upregulating circLRRC8A was rescued by miR-125a-3p (Fig. 3). In summary, we revealed a novel role for circLRRC8A in preventing GCs senescence by acting as a ceRNA of miR-125a-3p, resulting in the upregulation of NFE2L1. Like circLRRC8A, a handful of other circRNAs have been found to sponge microRNAs and are altered in an age-dependent manner across specific human organs and tissues [23, 34]. As reported, circDDX10 was highly expressed in human GCs, and it gradually decreased with aging and could act as a miRNA sponge to participate in the process of ovarian aging [35]. Another study demonstrated that caused by chronic exposure to UV, circCOL3A1-859,267 was downregulated in human dermal fibroblasts and regulated type I collagen expression by sequestering miR-29c for the prevention and treatment of photoaging [36]

CircLRRC8A is generated through back splicing of exon 3 of the gene that encodes LRRC8A, an mRNA component of the volume-regulated anion channel (VRAC). Whether LRRC8A plays a role in senescence, as we propose for circLRRC8A in the present study, is not known. Conversely, LRRC8A promotes proliferation in other cellular states, such as cancer. LRRC8A could promote the growth of gastric cancer cells via the p53 signaling pathway, and si-LRRC8A suppressed the proliferation and movement of gastric cancer cells and enhanced apoptosis [37]. The most important mechanism of circRNA cyclization is dependent on RBPs binding to introns upstream and downstream of the coding region. Protein quaking (QKI) enhances circRNA formation by binding to its recognition motif in introns flanking circRNA-forming exons [38]. In the present study, we showed that the RNA-binding protein EIF4A3 could bind to the upstream and downstream introns of circLRRC8A, promoting the cyclization of circLRRC8A. Further functional experiments demonstrated that the biogenesis of circRNA promoted by EIF4A3 played a crucial role in the BMSCs-Exos-mediated repair of ovarian function. As an important regulator of posttranscriptional regulation processes, including mRNA splicing, transport, translation, and surveillance [39], EIF4A3 could also bind to the pre-mRNAs of other circRNAs; for example, EIF4A3 could bind to circ-ZNF609 pre-mRNAs to form circular structures, and circ-ZNF609 expression was up or downregulated after promoting and inhibiting EIF4A3 expression [40].

MiR-125a-3p has been verified to induce cellular senescence and apoptosis and inhibit cancer migration and invasion by regulating certain pathways associated with cancer growth and by inhibiting oncogenic proteins. Silencing miR-125a-3p promotes myelin repair in models of white matter demyelination [41]. As the target of miR-125a-3p, NFE2L1 is a key factor that regulates the adaptive antioxidant response to oxidative stress. When oxidative stress occurs, NFE2L1 is exported to the cytoplasm from the endoplasmic reticulum (ER) and truncated by proteases to generate its transcriptionally active forms that enter the nucleus and bind ARE-dependent promoters. An analysis of human heart ventricles from 431 donors (age 20-79) in the GTEx database revealed that NFE2L1 expression was significantly decreased in the elderly demographic (age 60-79). NFE2L1 overexpression protects adult hearts from ischemia/reperfusion (I/R) injury by activating the expression of SOD1 and HMOX1 (ROS scavenger) [42]. In our study, silencing NFE2L1 aggravated oxidative injury and senescence in GCs, also hinting at its antiaging potential.

## Conclusion

In conclusion, our findings show that EIF4A3-induced BMSCs-derived exosomal circLRRC8A alleviates oxidative damage in GCs by regulating the miR-125a-3p/NFE2L1 axis. CircLRRC8A may serve as a novel potential diagnostic biomarker, and exosomal circLRRC8A may provide a promising therapeutic target for POF. Due to their biocompatibility and nonimmunogenicity, Exos may be a promising strategy to be an alternative cell-free approach [43]. Exos-circLRRC8A can be found in plasma and follicular fluid, highlighting its possible application in diagnosis as well as a novel therapy. Despite the promising prospects, further studies are lacking compared to studies on mRNAs and miRNAs, which means that before clinical application, we need a more accurate understanding of circRNAs [44].

## Supplementary Information

ESM 1 (DOCX 10068 kb)

## Data Availability

Circular RNA expression profiling of human granulosa cells and miRNA profile of the human follicular fluids in young and advanced-aged women were obtained from the GEO database. The GEO accession numbers are GSE97193 and GSE63737.

## Abbreviations

*POF*: Premature ovarian failure
*MSCs*: Mesenchymal stromal cells
*MSCs-Exos*: Mesenchymal stromal cells-derived exosomes
*HEK293*: Human embryonic kidney 293
*KGN*: Human granulosa-like tumor cells line
*GCs*: Granulosa cells
*circRNAs*: Circular RNAs
*miRNAs*: MicroRNAs
*qRT-PCR*: Quantitative RT-PCR
*WB*: Western blot analysis
*EIF4A3*: Eukaryotic initiation factor 4A3
*RBPs*: RNA-binding proteins
*NFE2L1*: Nuclear factor erythroid-2-related factor 1
*TEM*: Transmission electron microscopy
*NTA*: Nanoparticle tracking analysis
*ceRNA*: Competitive endogenous RNA
*P21*: Cyclin-dependent kinase inhibitory protein-1
