# Supplementary material for: EIF4A3-Induced Exosomal circLRRC8A Alleviates Granulosa Cells Senescence Via the miR-125a-3p/NFE2L1 axis
Source: Stem Cell Rev Rep. 2023 May 27;19(6):1994–2012. doi: 10.1007/s12015-023-10564-8 (PMC10390409; doi:10.1007/s12015-023-10564-8)
Supplement: Supplementary file 1 — (DOCX 10068 kb) [file 12015_2023_10564_MOESM1_ESM.docx]

Supplementary Materials for

EIF4A3-induced exosomal circLRRC8A alleviate granulosa cells senescence via the miR-125a-3p/NFE2L1 axis

Jie Xing+, Mengxue Zhang+, Shijie Zhao, Mingjun Lu, Li Lin, Lu Chen, Wujiang Gao, Lu Chen, Wenxin Li, Junyu Shang, Jiaming Zhou, Xiaolan Zhu*.

Correspondence to: [zxl2517@163.com](mailto:zxl2517@163.com).

**This PDF file includes:**

Figures. S1 to S6

Tables S1

**Figure. S1**

**
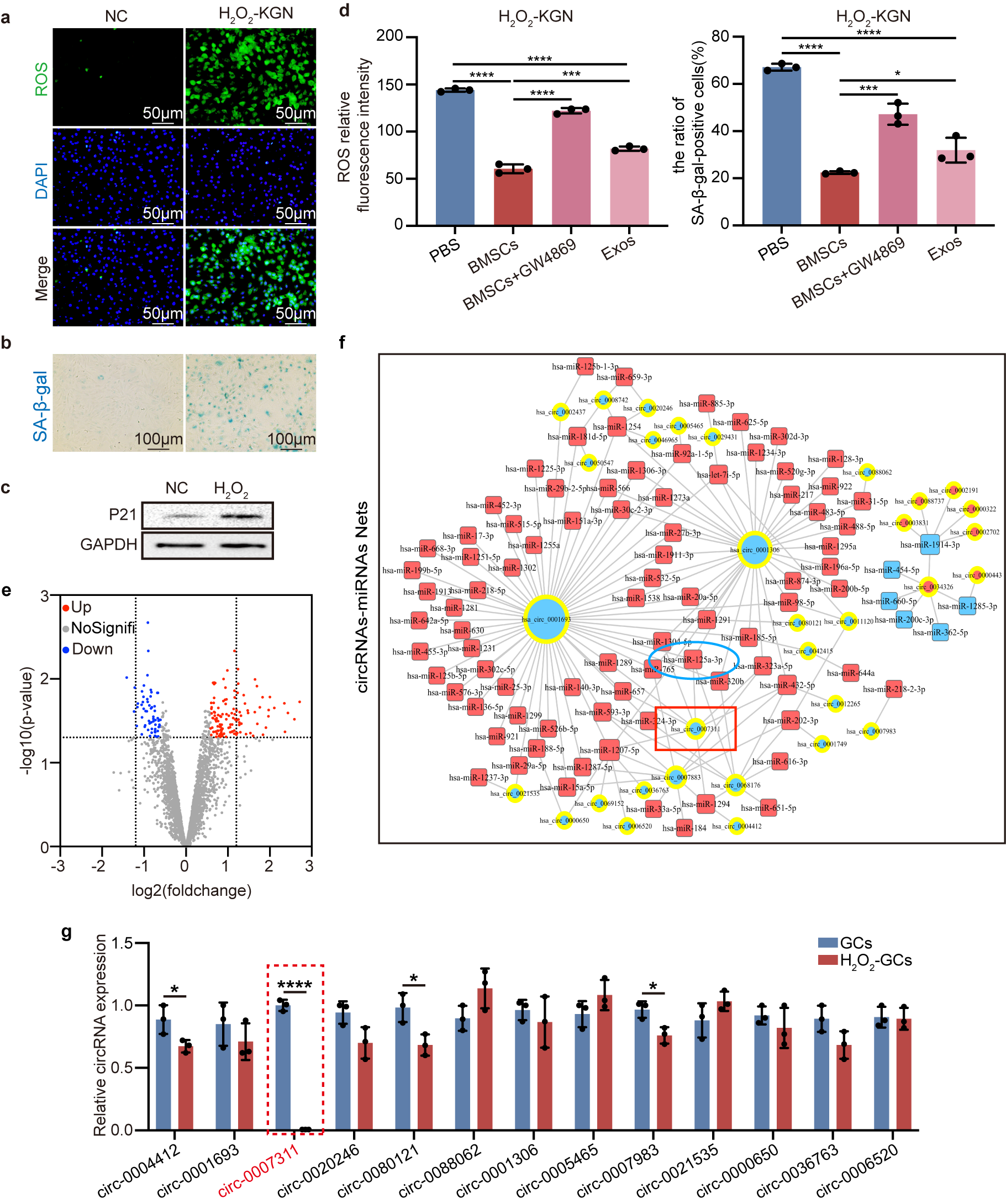
**

**Supplementary Fig. 1** CircLRRC8A is significantly upregulated in oxidatively damaged GCs treated with BMSCs-derived exosomes **a** The reactive oxygen species levels were detected by DCFH-DA staining in the KGN cells and H_2_O_2_-KGN cells. **b** SA-β-gal staining in the KGN cells and H_2_O_2_-KGN cells. **c** Relative protein levels of P21 in KGN cells and H_2_O_2_-KGN cells. **d** The ROS staining and SA-β-gal staining in the PBS, BMSCs, BMSCs +GW4869, and Exos groups (n=3). **e** Volcano plot of differentially expressed circRNAs. **f** Networks of circRNAs-miRNAs. Round represent circRNAs and square represent miRNAs. **g** The expression of the top 13 differentially low expressed circRNAs were verified in granulosa cells and granulosa cells with H_2_O_2_ treatment by qRT-PCR (n=3).

**Figure. S2**


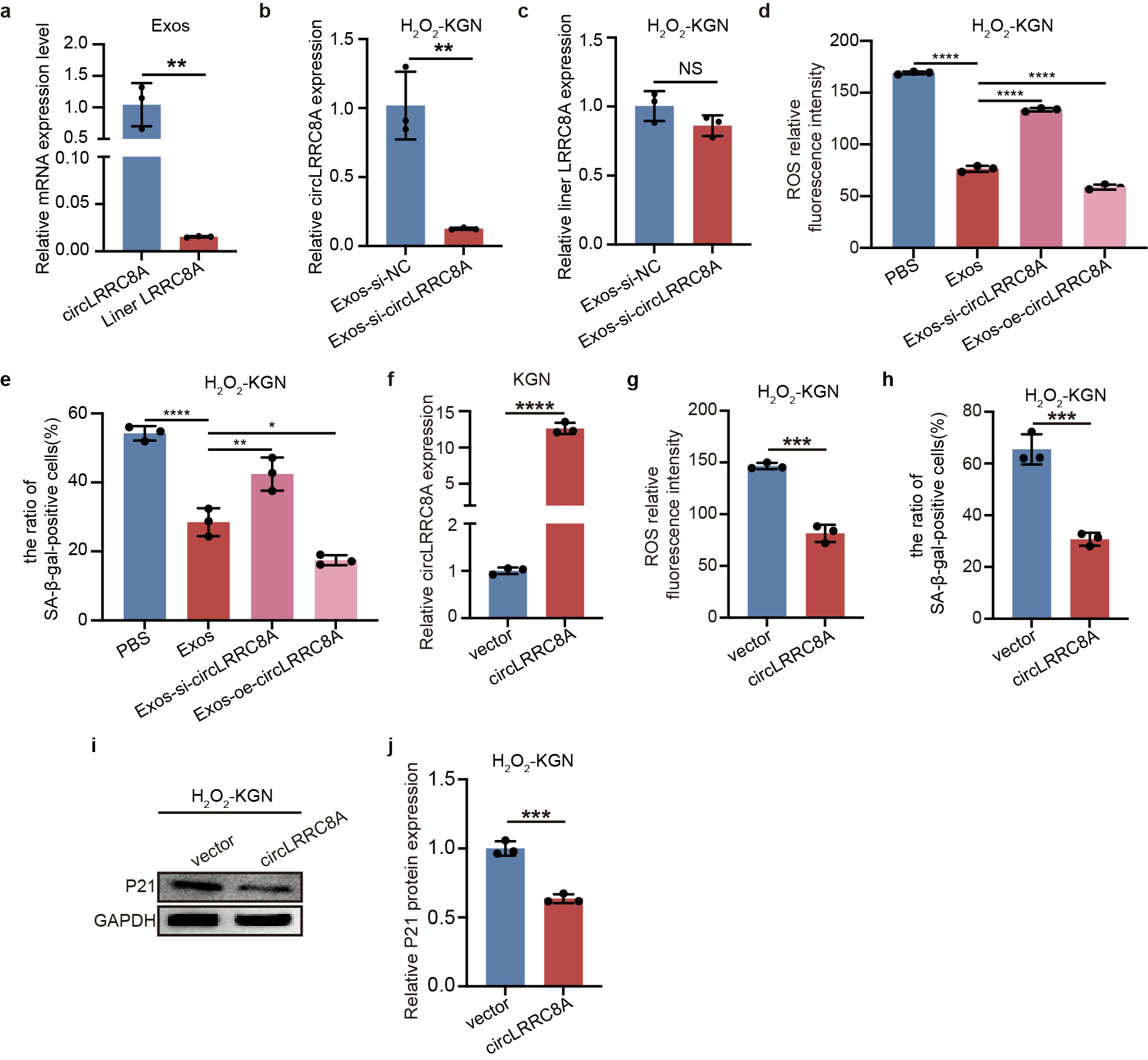


**Supplementary Fig. 2** BMSCs-Exos rescued oxidative damage in KGN cells by releasing circLRRC8A. **a** a Relative expression of liner LRRC8A was tested by qRT-PCR in Exos (n=3). **b, c** Relative expression of circLRRC8A and liner LRRC8A in H_2_O_2_-KGN cells when cocultured with exosomes secreted from BMSCs transfected with siRNA or si-NC (n=3). **d, e** The ROS staining and SA-β-gal staining in H_2_O_2_-induced KGN cells when cocultured with exosomes secreted from BMSCs transfected with siRNA or overexpression plasmid of circLRRC8A (n=3). **f** KGN cells were transfected with circLRRC8A plasmid for 24h, then relative circLRRC8A expression tested by qRT-PCR (n=3). **g** ROS levels of H_2_O_2_-induced KGN cells transfected by oe-circLRRC8A (n=3). **h** SA-β-gal staining assays in H_2_O_2_-KGN cells transfected by vector plasmid and circLRRC8A plasmid (n=3). **i, j** Relative protein levels of P21 in H_2_O_2_-KGN cells transfected with vector plasmid and circLRRC8A plasmid (n=3).

**Figure. S3**

**
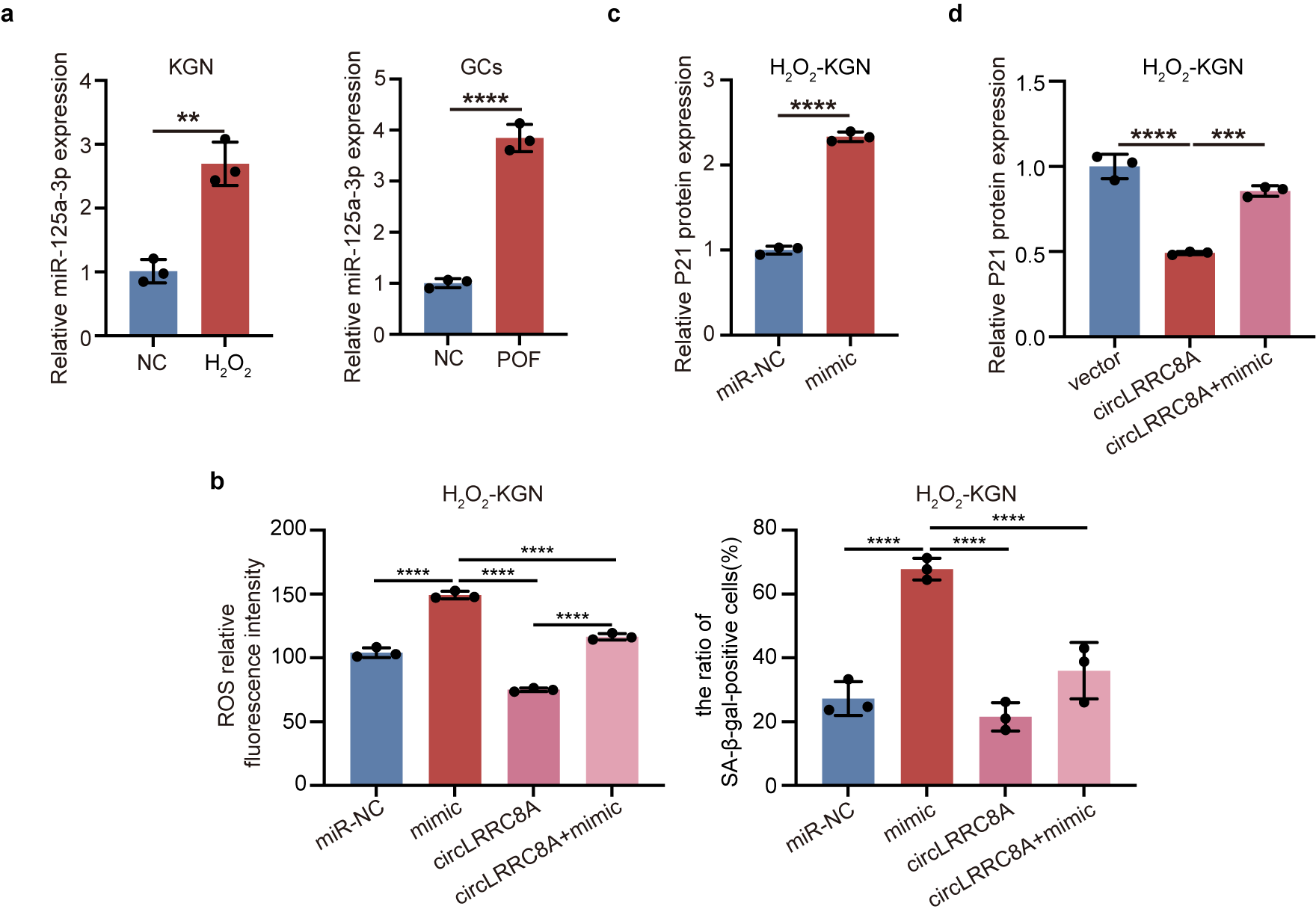
**

**Supplementary Fig. 3** circLRRC8A regulates GCs senescence caused by oxidative damage by inhibiting miR-125a-3p. a Relative expression of miR-125a-3p was tested by qRT-PCR in KGN cells treated with H_2_O_2_ or the primary human granulosa cells obtained from POF patient (n=3). b ROS staining and SA-β-gal staining was performed for oxidative damage and senescence of H_2_O_2_-KGN cells (n=3). c Western blot assays showing the expression of P21 in H_2_O_2_-KGN cells transfected with miR-NC or miR-125a-3p mimic (n=3). d Western blot showing the expression levels of P21 in H_2_O_2_-KGN cells transfected with vector plasmid, circLRRC8A plasmid, or cotransfected with miR-125a-3p mimic and circLRRC8A plasmid (n=3).

**Figure. S4**


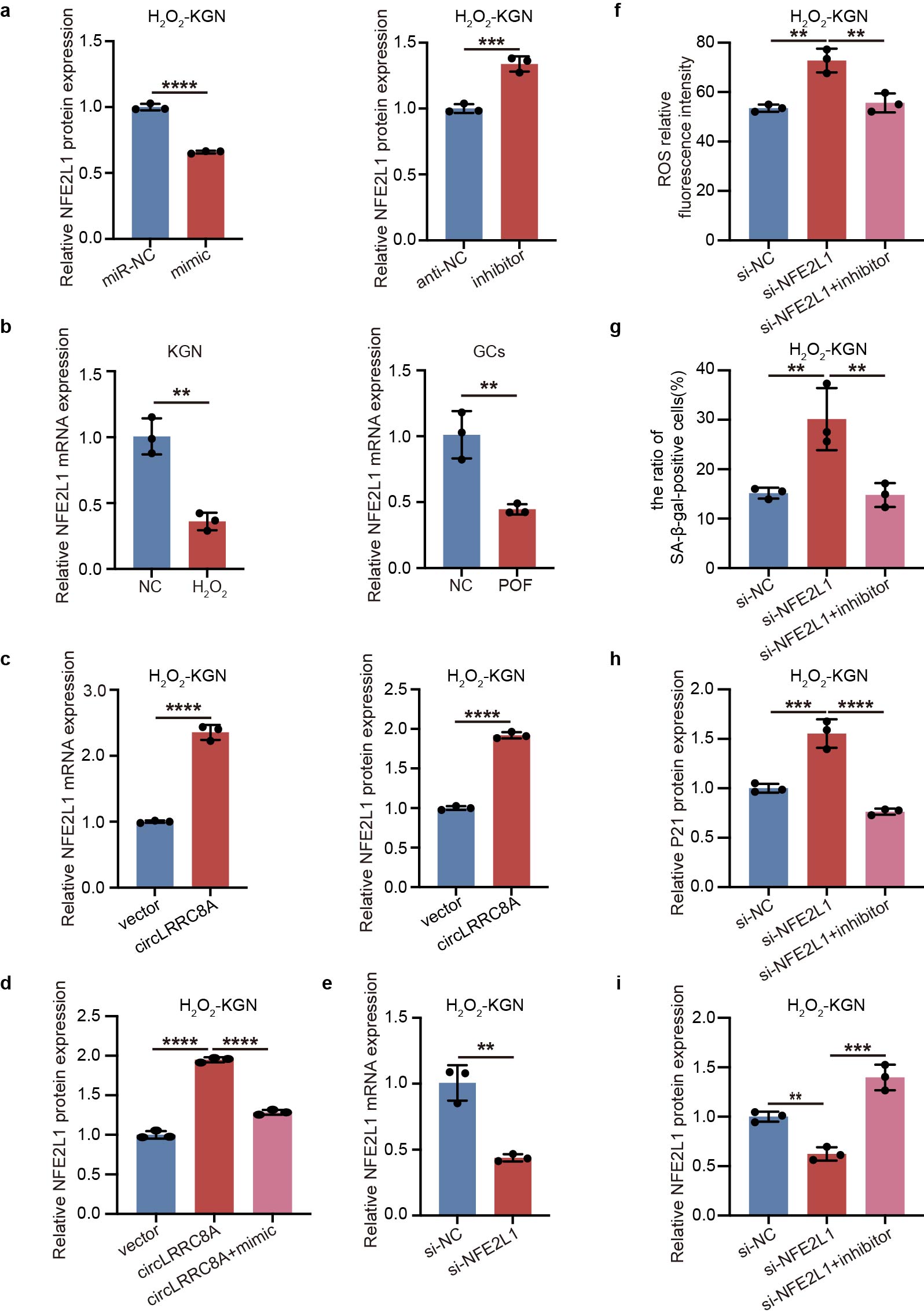


**Supplementary Fig. 4** CircLRRC8A upregulates NFE2L1 expression by sponging miR-125a-3p to suppresses GCs senescence. **a** Western blot assays showing the expression of NFE2L in H_2_O_2_-KGN cells transfected with miR-125a-3p mimic or miR-125a-3p inhibitor (n=3). **b** Relative expression of NFE2L1 in normal granulosa cells and senescent granulosa cells (n=3). **c** Overexpression of circLRRC8A significantly upregulate the expression levels of NFE2L1 in H_2_O_2_-KGN cells (n=3). **d** Western blot showing the expression levels of NFE2L1 in H_2_O_2_-KGN cells transfected with vector plasmid, circLRRC8A plasmid, or cotransfected with miR-125a-3p mimic and circLRRC8A plasmid (n=3). **e** Silencing of NFE2L1 significantly downregulate the expression levels of NFE2L1 in senescent-KGN cells (n=3). **f, g** ROS staining and SA-β-gal staining of H_2_O_2_-KGN cell transfected by si-NC, si-NFE2L1, or cotransfected by miR-125a-3p inhibitor and si-NFE2L1 (n=3). **h** The protein levels of P21 in H_2_O_2_-KGN cells transfected with si-NC, si-NFE2L1, or cotransfected with miR-125a-3p inhibitor and si-NFE2L1 (n=3). **i** Western blot assays showing the expression levels of NFE2L1 in H_2_O_2_-KGN cells transfected with si-NC, si-NFE2L1, or cotransfected with miR-125a-3p inhibitor and si-NFE2L1 (n=3).

**Figure. S5**


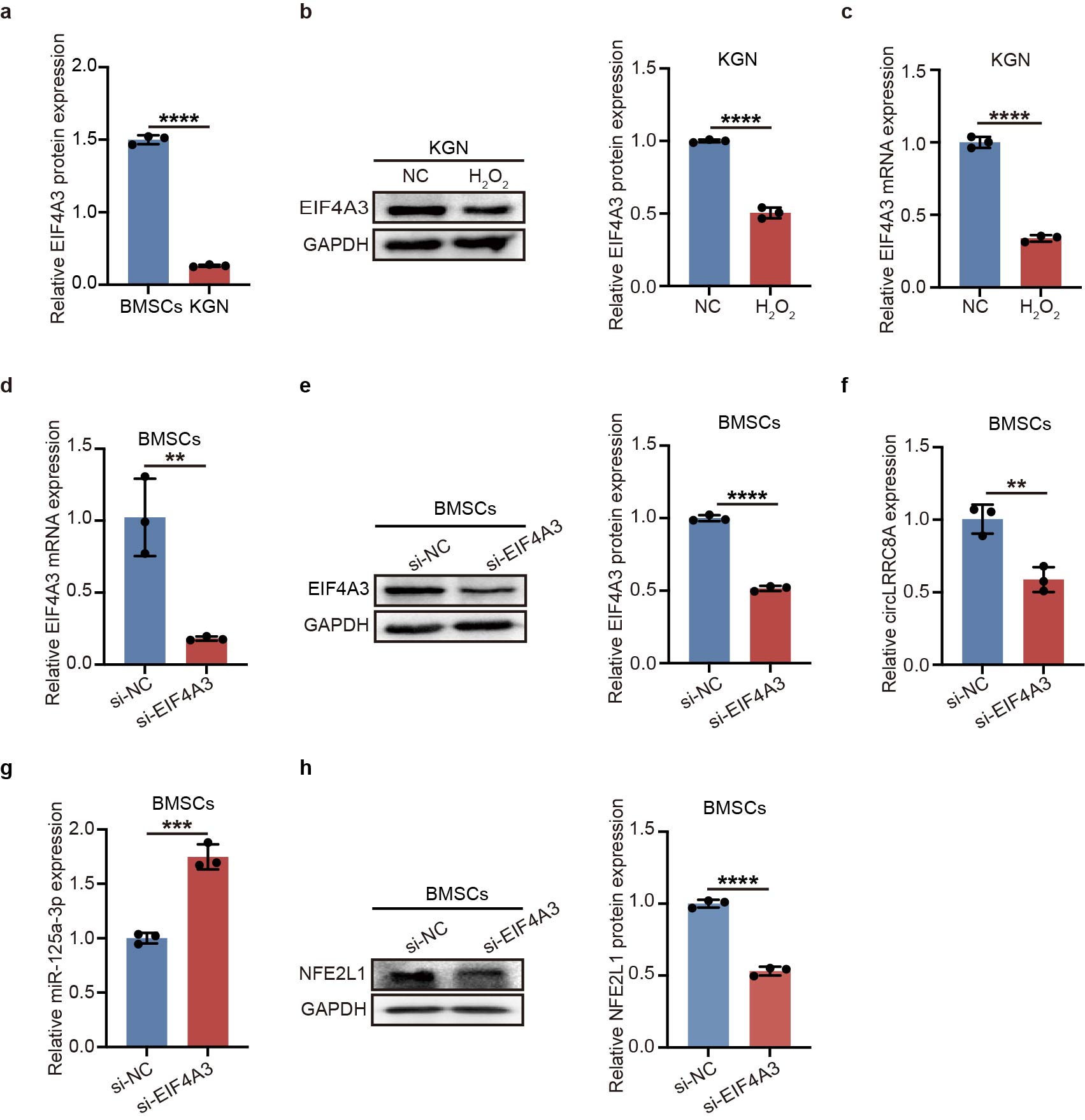


**Supplementary Fig. 5** RNA binding protein EIF4A3 facilitates the biogenesis of circLRRC8A. **a** The protein levels of EIF4A3 in BMSCs and KGN cells (n=3). **b, c** The levels of mRNA and protein of EIF4A3 in H_2_O_2_-KGN cell (n=3). **d, e** The effects of transfection with si-EIF4A3 on EIF4A3 in BMSCs (n=3). **f, g** The level of circLRRC8A and miR-125a-3p in BMSCs treated with EIF4A3 siRNA (n=3). **h** The effects of transfection with si-EIF4A3 on NFE2L1 expression in BMSCs (n=3).**Figure. S6**


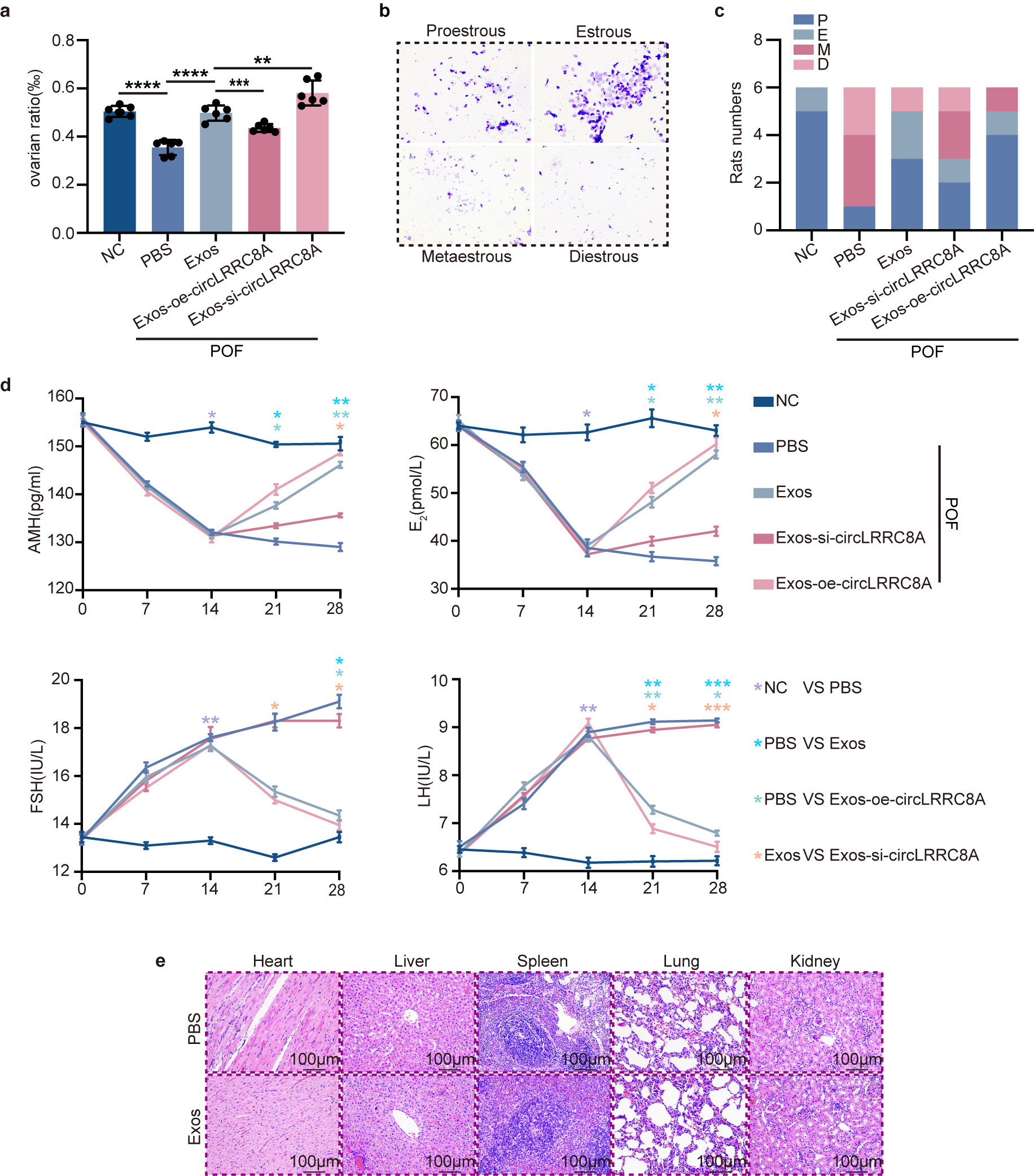


**Supplementary Fig.6** Exos-circLRRC8A regulate GCs senescence in POF in *vivo*. **a** Ovarian/body weight ratio (n=6). **b** Crystal violet staining of vaginal smears. **c** Compositions of estrous cycle in different groups (n=6) (P, proestrous; E, estrous; M, metestrous; D, diestrous). **d** Serum hormones levels for AMH, E2, FSH, and LH (n=6). **e** Histopathology of heart, liver, spleen, lung, kidney from rat treated by Exos.

**Table S1**

**Primer sequences**

| Genes | Forward (5'-3') | Reverse (5'-3') | Organism |
| --- | --- | --- | --- |
| circLRRC8A | TACCTGGACCTCAGCCACAA | GCTCTGTCACCGGAATCATGG | Human |
| Convergent primers | GTTGAACCATGATTCCGGTGACAGAG | CCGGTTGGCCGTGATGGC | Human |
| divergent primers | CCCATCCAGATCGGCAACCT | GCAGCTTGCGGCAGTAGAAG | Human |
| miR-125a-3p | acaggtgaggttcttgggagc |  | Human |
| NFE2L1 | GCAGCCAGGACTTCTTACTCTTCAG | TGGAGCCGAAGGTGGAGTTGAG | Human |
| sequence A | AGGTTGAACCATGATTCCGGTG | GGATCCGGTATGCTGGCTG | Human |
| sequence B | GGTGGTCTGTGGGTGCCA | CTTACCTGGCTGGGGAAG | Human |
| EIF4A3 | CAGGGCGTGTTTTTGATATGAT | ATCAGCTTCATCCAAAACCAAC | Human |
| circLRRC8A | AGGCGAAGGCACTGTTTGAGAAG | CACGGTGTAGCAGATGATGAGGAAG | Rat |
| miR-125a-3p | ACAGGTGAGGTTCTTGGGAG |  | Rat |
| NFE2L1 | GGAAGGAGATGGTGTGGAGGAGAG | ATGAGGAGCCCAGGTGTTAGTAGG | Rat |
